# Neuronal STING activation in amyotrophic lateral sclerosis and frontotemporal dementia

**DOI:** 10.1007/s00401-024-02688-z

**Published:** 2024-03-13

**Authors:** Christine Marques, Aaron Held, Katherine Dorfman, Joon Sung, Catherine Song, Amey S. Kavuturu, Corey Aguilar, Tommaso Russo, Derek H. Oakley, Mark W. Albers, Bradley T. Hyman, Leonard Petrucelli, Clotilde Lagier-Tourenne, Brian J. Wainger

**Affiliations:** 1 Department of Neurology, Sean M. Healey & AMG Center for ALS, Massachusetts General Hospital, Boston, MA USA; 2 Harvard Medical School, Boston, MA USA; 3 Department of Pathology, Massachusetts General Hospital, Boston, MA USA; 4 Alzheimer Disease Research Unit, Department of Neurology, Massachusetts General Hospital, Charlestown, MA USA; 5 Department of Neuroscience, Mayo Clinic, Jacksonville, FL USA; 6 Broad Institute of Harvard University and MIT, Cambridge, MA USA; 7 Department of Anesthesiology, Critical Care and Pain Medicine, Massachusetts General Hospital, Boston, MA USA; 8 Harvard Stem Cell Institute, Cambridge, MA USA

**Keywords:** Motor neuron disease, Amyotrophic lateral sclerosis, DNA damage, Neuroinflammation, STING, Central neurons

## Abstract

The stimulator of interferon genes (STING) pathway has been implicated in neurodegenerative diseases, including Parkinson’s disease and amyotrophic lateral sclerosis (ALS). While prior studies have focused on STING within immune cells, little is known about STING within neurons. Here, we document neuronal activation of the STING pathway in human postmortem cortical and spinal motor neurons from individuals affected by familial or sporadic ALS. This process takes place selectively in the most vulnerable cortical and spinal motor neurons but not in neurons that are less affected by the disease. Concordant STING activation in layer V cortical motor neurons occurs in a mouse model of C9orf72 repeat-associated ALS and frontotemporal dementia (FTD). To establish that STING activation occurs in a neuron-autonomous manner, we demonstrate the integrity of the STING signaling pathway, including both upstream activators and downstream innate immune response effectors, in dissociated mouse cortical neurons and neurons derived from control human induced pluripotent stem cells (iPSCs). Human iPSC-derived neurons harboring different familial ALS-causing mutations exhibit increased STING signaling with DNA damage as a main driver. The elevated downstream inflammatory markers present in ALS iPSC-derived neurons can be suppressed with a STING inhibitor. Our results reveal an immunophenotype that consists of innate immune signaling driven by the STING pathway and occurs specifically within vulnerable neurons in ALS/FTD.

**Supplementary Information:**

The online version contains supplementary material available at 10.1007/s00401-024-02688-z.

## Introduction

Neuroinflammation, characterized largely by the initiation and propagation of innate immune signaling, leads to the activation of microglia and astrocytes within the central nervous system and plays an increasingly appreciated role in neurodegenerative diseases [36]. Current understanding posits that microglia and astrocytes orchestrate a neuroinflammatory response, either in direct response to exogenous neuronal damage or through an autonomous inflammatory capacity. Recent studies have implicated the STING pathway, a primary link between foreign or damaged DNA and innate immune activation [11], in neuroinflammation and neurodegenerative diseases [76]. Cytoplasmic double-stranded DNA, recognized by the DNA sensor cyclic GMP-AMP synthase (cGAS), leads to 2′3′-cyclic GMP-AMP (cGAMP) synthesis, activation of the canonical STING–TBK-1–IRF3 pathway, and downstream type-I interferon signaling. Damaged nuclear DNA can also trigger the non-canonical STING pathway, which operates independently of cGAS and results in the predominant activation of NF-κB [20, 39]. Increased STING signaling occurs in Parkinson’s disease [89] and amyotrophic lateral sclerosis (ALS) [62, 102], for which global STING knockout reduced disease progression in mouse models [62, 89, 102]. Such benefits have been thought to result from STING elimination in glial and myeloid cells [76], consistent with the reduction in interferon markers observed after blocking STING in myeloid cells from individuals with ALS [62]. Because the accumulation of DNA damage within neurons is a primary feature of ALS, frontotemporal dementia (FTD), and other dementias [14, 21, 60], we hypothesized that STING may initiate an innate immune response within neurons themselves. We focused on the spectrum of ALS/FTD on account of the central role of DNA damage at the convergence of disease mechanisms.

ALS is a devastating and rapidly fatal degenerative disease of the motor nervous system [10], and it shares pathological features with about 50% of FTD cases [5]. While approximately 90% of ALS cases are sporadic (sALS), 10% are familial (fALS) and result from a mutation in one of more than 30 genes that span a broad range of biological functions. These variants include *C9orf72* intronic repeat expansion, the most common familial cause of both ALS and FTD and a major contributor to sporadic disease as well [24]. The roles of many ALS genes implicate DNA damage as an overarching mechanism, particularly *C9orf72*, *FUS*, and *TARDBP* [46], and postmortem analysis of laser-captured cortical and spinal motor neurons from sALS subjects reveals upregulation of DNA damage and response pathways [42–44].

In this study, we established that STING activation occurs in vulnerable cortical and spinal motor neurons in both fALS and sALS but not in less affected neurons. Leveraging parallel approaches across postmortem tissue from multiple fALS variants and sALS, mouse models of C9orf72 repeat expansion disease, and neurons derived from human iPSCs harboring fALS mutations, we observed a consistent activation of STING and its downstream effectors. The parallel results in iPSC-derived neurons suggest that STING activation in neurons occurs in a neuron-autonomous manner, with DNA damage serving as a key mechanism. Supporting the importance of STING signaling in neuronal inflammation in ALS, STING inhibition blocks inherent innate immune markers present in fALS iPSC-derived neurons.

## Materials and methods

All experiments were conducted in accordance with relevant guidelines and followed protocols approved by the Massachusetts General Hospital Institutional Review Board, Institutional Animal Care and Use Committee, and Partners Institutional Biosafety Committee. Studies performed in postmortem human tissue were approved by the Massachusetts Alzheimer’s Disease Research Center (ADRC) and the Veterans Affairs Biorepository Brain Bank (VABBB).

### Analysis of publicly available ALS datasets

FASTQ files (GSE143743 [1] and GSE76220 [49]) were obtained from the NCBI gene expression omnibus (GEO) database or were previously generated by our lab (dbGAP: phs002440.v2.p1 [35]). FASTQ files were aligned to GRCh38 using STAR (2.7.3a) [19], and read counts were generated using –quantMode GeneCounts in STAR. Differentially expressed genes were determined using DESeq2 (1.36.0) [57] in R (4.0.3). For the reanalysis of postmortem sALS SMNs [49], a model of design =  ~ Patient Sex + Disease was used to normalize for effects by patient sex as in [35]. Genes with adjusted p-value < 0.05 met statistical significance for differential expression. Gene set enrichment analysis (GSEA) (fgsea 1.22.0) [92] was performed in R (4.2.1) using log2(fold change) values.

### Postmortem human and mouse tissue immunohistochemistry and immunostaining

Postmortem motor cortex tissue included sections from *C9orf72* repeat expansion with ALS (five ALS alone and three ALS/FTD), p-TDP-43-negative AD (8), and non-neurological disease controls (6) provided by the ADRC, as well as TDP-43, FUS, PFN1, KIF5A, NEK1 fALS, and four non-neurological controls provided by the VABBB. Information on human samples is provided in Supplementary Table 1. Briefly, formalin-fixed, paraffin-embedded (FFPE) slides from postmortem human primary motor cortex and occipital cortex were de-paraffinized with xylene followed by a descending ethanol series. Peroxidase quenching was performed by incubating slides in 3% hydrogen peroxide solution for 30 min. Sections were rinsed with PBS, and antigen retrieval was then performed by microwaving sections in 10 mM citrate buffer, pH 6.0, and allowing them to cool on ice for 30 min. The sections were permeabilized using 0.4% Triton X-100 (Millipore Sigma, 9400) for 8 min at room temperature (RT) and incubated with a blocking solution containing 2.5% normal horse serum (Vectastain ABC kit) for 90 min at RT. The sections were then incubated with primary antibody anti-STING (1/50, R&D systems, AF6516) overnight at 4 °C. Immunolabeling was then visualized using the biotinylated secondary antibody sheep IgG horseradish peroxidase (HRP) for 90 min at RT and incubated with the avidin–biotin enzyme complex for 30 min. Stains were visualized by incubation with the Vector peroxidase substrate kit DAB (VectorLabs). Slides were then dehydrated in ascending ethanol/xylene series and coverslipped with Cytoseal X. Immunohistochemical negative controls included incubation of sections with no primary or secondary antibody but with all other immunoperoxidase-DAB steps unchanged. Investigators were blinded throughout the processing and analysis of IHC staining. Imaging of immunoperoxidase sections was done using a 40 × objective on a Nanozoomer.

For *C9orf72* mouse tissue, FFPE sagittal brains from four AAV (G_4_C_2_)_2_ (two males and two females) and five AAV (G_4_C_2_)_149_ (three males and two females) 1-year-old mice were sliced into 6 μm sections using a microtome. For STING antibody validation, 1-year-old wild-type (WT) and goldenticket (gt) STING^gt/gt^ mice were transcardially perfused with cold 0.01 M PBS, followed by cold 4% PFA in 0.01 M PBS. Their colon and spleen were collected and cut on a vibratome into coronal sections of 40 μm.

For tissue immunofluorescence staining, FFPE human and mouse sections were de-paraffinized with xylene and re-hydrated in descending ethanol series. Antigen retrieval was then performed by microwaving sections in 10 mM citrate buffer, pH 6.0, and allowing them to cool on ice for 30 min. Sections were permeabilized using 0.4% Triton X-100 (Millipore Sigma, 9400) for 8 min at RT and incubated with a blocking solution containing 10% Normal Donkey Serum and 0.1% Triton X-100 for 90 min at RT. Human sections were then incubated with primary antibodies including anti-STING (1/50, R&D systems, AF6516), anti-CRYM (1/250, Abcam, ab220085), anti-p-IRF3 (1/50, Cell Signaling Technology, 29047S), and anti-HuC/HuD (1/2000, Sigma-Aldrich, MABN153) overnight at 4 °C. Mouse sections were incubated with primary antibodies including anti-STING (1/100, Proteintech, 19,851–1-AP), anti-p-IRF3 (1/50, Cell Signaling Technology, 29047S), anti-CTIP2 (1/100, Abcam, ab18465), and anti-p-NF-κB (1/50, Santa Cruz Biotechnology, sc-136548) overnight at 4 °C (Supplementary Table 2). After three washes with PBS, the slides were incubated with species-specific Alexa Fluor (− 488, − 647, − 568; Thermo Fisher Scientific) secondary antibodies at a 1/500 dilution for 90 h at RT. Finally, the slides were incubated for 5 min with DAPI solution (Thermo Fisher Scientific, D1306) for nuclear staining and washed three times in PBS. After 5 min incubation in 70% ethanol, the sections were immersed in Autofluorescence Eliminator Reagent (Millipore, 2160) for 5 min. The sections were coverslipped with ProLong Diamond Antifade solution (Life Technologies, P36962). Confocal images were taken using a 20 × objective on a Zeiss LSM 900 Confocal microscope. Investigators were blinded throughout the processing and quantitative analysis of immunofluorescence staining of CTIP2-positive and STING-positive neurons in mouse sections.

### Human iPSCs

Human iPSC lines were obtained from the Target ALS Repository, Harvard University, Jackson Laboratory, and the NINDS Human Cell and Data Repository (NHCDR). Human iPSC lines included non-isogenic control lines 11a [9], ND50003 (FA0000010) and KOLF2.1 J [75] and five fALS lines harboring mutations in C9orf72: 19f [45], NDS00268 (ND50074), NDS00269 (ND50075), NDS00270 (ND50076), and NDS00273 (ND50080). An isogenic pair of lines consisting of an unedited control TDP-43^+/+^ line and an edited TDP-43^+/G298S^ iPSC line (harboring a single fALS mutation) was previously generated [77]. iPSCs were cultured as described previously [65]. Briefly, iPSCs were maintained on Matrigel-coated 6 well-plates (Corning, 354,277, and Falcon, 353,046) in mTeSR media (Stemcell Technologies, 85,850) for SMN differentiation or mTeSR Plus media (Stemcell Technologies, 100–0274) for NGN2 induction at 37 °C with 5% CO_2_.

### Cell culture

#### Primary mouse cortical neurons

Primary cortical neurons were isolated from cerebral cortices of embryonic day 13–14 mouse embryos (Charles River Laboratories) as previously described [93] with modifications. Briefly, pregnant female mice were euthanized between embryonic days 13 and 14, and embryonic cortices were dissected and enzymatically dissociated using a papain dissociation kit (Worthington Biochemical, LK003178). Neurons were plated on poly-D-lysine-coated 384- (Corning Life Sciences, 354,663) and 24-well plates (Falcon, 08-772-1) at densities of 1 × 10^4^ and 2.5 × 10^5^ viable neurons, respectively. The neurons were maintained at 37 °C with 5% CO_2_ in Neurobasal medium (Life Technologies, 21,103–049) supplemented with 1% GlutaMAX (Thermo Fisher Scientific, 35,050–061), 2% B-27 supplement (Life Technologies, 17,504–044), and 1% penicillin/streptomycin (Life Technologies, 15,070–063). Primary cortical neurons were grown for 5 days before receiving described drug treatments and analysis.

#### SMN differentiation from iPSCs

SMN differentiation from iPSCs followed a previously described protocol [87] with modification [65]. Briefly, iPSCs were enzymatically dissociated into single cells for 10 min at 37 °C with StemPro Accutase Cell Dissociation Reagent (Thermo Fisher Scientific, A1110501) and counted using a Countess II FL Automated Cell Counter (Thermo Fisher Scientific, AMQAF1000). Cells were neuralized as a suspension culture at a density of 2 × 10^6^ cells/ml using SB431542 (20 µM, Sigma-Aldrich, S4317), LDN-193189 (0.1 µM, Stemgent 040019), CHIR-99021 (3 µM, Tocris, 99,021), FGF2 (10 ng/mL, Thermo Fisher Scientific, 13,256,029), and ascorbic acid (10 µM) (Sigma, A4403) in N2/B27 media (1:1 mixture of Neurobasal and Advanced DMEM/F12 (Life Technologies, 12,634–028) supplemented with 1% GlutaMAX, 100 µM β-mercaptoethanol (Life Technologies, 31,340–010), 2% B27-supplement, 1% N2-supplement (Gibco, 17,502–048), and 1% penicillin/streptomycin. On day 2, neural spheres were patterned to spinal cord identity by treating with retinoic acid (0.1 µM, Sigma, R2625), smoothened agonist (SAG, 500 nM, EMD Calbiochem, 566,660), along with SB-431542, LDN-193189, and CHIR-99021 in N2/B27 media for an additional 5 days. On day 7, brain-derived neurotrophic factor (BDNF, 10 ng/ml, Life Technologies, PHC7074) was added to the N2/B27 media to generate SMN progenitors. On day 9, SMN progenitors were cultured in the previous media and additionally supplemented with DAPT (10 µM, Tocris Bioscience, 2634) for 5–7 days. After 16 total days in culture, embryoid bodies were dissociated using 0.05% Trypsin–EDTA, pre-mixed with Matrigel, and seeded on poly-D-lysine-coated plates at either 1 × 10^4^ cells per well (384-well plates, immunostaining) or 2.5 × 10^5^ cells per well (24-well plates, RNA). The following day, the neurons were treated with uridine/5-fluoro-deoxyuridine (U/FDU, 1 µM) to remove residual proliferating cells and cultured in Neurobasal media supplemented with 1% GlutaMAX, 1% non-essential amino acids (Corning 25–025-CI), ß-mercaptoethanol (100 µM), B27-supplement (1%), N2-supplement (1%), retinoic acid (1 µM), ascorbic acid (2.5 µM), BDNF (10 ng/ml), glial-derived neurotrophic factor (GDNF, 10 ng/ml, Life Technologies PHC7044), ciliary neurotrophic factor (CNTF, 10 ng/ml, Life Technologies PHC7015), insulin-like growth factor (IGF-I, 10 ng/ml, R&D systems 291-G1-200), and penicillin/streptomycin (1%). Media was changed every 2–3 days.

#### NGN2 induction from iPSCs

NGN2 neurons were generated using a PiggyBac strategy [35, 75, 103]. PiggyBac plasmids were generated with tet-inducible transcription factors NGN2 [104] and nucleofected into iPSC lines together with a PiggyBac transposase plasmid using the Nucleofector I (Lonza, A-23) and the Human Stem Cell Nucleofector Kit 1 (Lonza, VPH-5012). Cells were plated on Vitronectin XF (Stemcell Technologies, 07180) and maintained in mTeSR Plus media supplemented with CET (Chroman1 (50 µM, MedChem Express, HY-15392), Emricasan (5 mM, Selleckchem, S7775), and Trans-ISRIB (0.7 mM, Tocris, 5284)) [12] for 24 h and selected using puromycin (10ug/mL, InvivoGen, ant-pr-1) for 48 h based on the presence of nuclear BFP2 signal, indicative of donor plasmid integration.

Selected iPSCs were then differentiated into post-mitotic neurons. In brief, 12 million iPSCs were plated in a T175 flask coated with Matrigel and cultured using induction media (DMEM/F12 medium, Life Technologies, 11,320–082; 1% N2-supplement; 1% non-essential amino acids; 1% GlutaMAX; and 1% penicillin/streptomycin) supplemented with CET and doxycycline (2 μg/mL, Sigma Aldrich, D9891-1G). A full media change was performed on day 2. On day 3, differentiated NGN2 neurons were dissociated using Accutase and frozen (1 million cells/vial) in induction media, supplemented with 40% FBS [Hyclone, SH30910.03HI) and 10% DMSO (Sigma, D2650)].

For individual experiments, NGN2 neurons were pre-mixed with Matrigel and plated in poly-D-lysine-coated plates, at either 10,000 cells/well (384-well plate, immunostaining) or 250,000 cells per well (24-well plate, RNA) in induction media supplemented with CET. After 24 h, the neurons were maintained at 37 °C with 5% CO_2_ in Neurobasal supplemented with 2% B27-supplement, NaCl (50 mM), 1% GlutaMAX, NT-3 (10 μg/mL, PeproTech, 450–03), BDNF (10 μg/mL), and 1% penicillin/streptomycin. The following day, the neurons were cultured in long-term media supplemented with aphidicolin (5uM, Cell Signaling Technology, 32,774). After 2 days, media was exchanged with fresh long-term media without aphidicolin. Every 3 days, 50% of the media was replaced with fresh media and maintained up to day 30 in culture. The neurons were grown for 10 to 30 days for time course analyses and for 20 days before receiving drug treatments.

### Lentiviral shRNA neuronal transduction

Two separate lentiviral shRNAs directed against TDP-43 and a scrambled shRNA control were obtained from Origene (TL308946V). For shRNA lentiviral particle transduction, isoTDP-43^+/+^ NGN2 neurons were incubated for 4 h with media containing lentivirus particles for GFP-shScramble or GFP-shTDP-43 and cultured for 48 h. Transduction efficiency was assessed by GFP expression. Neuronal cultures were analyzed for STING and γH2AX levels 48 h post-transduction.

### DNA-damaging agents and STING modulators

For induction of DNA damage, cells were incubated with etoposide (Sigma, E1383) and glutamate (Sigma, G1251) at 5 µM and 10 µM, respectively. For STING-activation experiments, 2′3′-cGAMP (Sigma, 5,318,890,001) was used at 20 μM in human iPSC-derived neurons, and DMXAA (Sigma, D5817) was used at 20 μg/ml in primary mouse cortical neurons. For STING-inhibition experiments, 1 μM H151 (Invitrogen, inh-h151) or 10 μM RU.521 (Sigma, SML2347) was used.

### RT-qPCR

RNA was extracted from cells using Trizol reagent (Life Technologies, 15,596–018) and purified according to the manufacturer’s instructions. Purified RNA was reverse transcribed using the High-Capacity cDNA Reverse Transcription Kit (Thermo Fisher Scientific, 4,368,814) according to the manufacturer’s instructions. RT-qPCR was carried out using iQ™ SYBR Green Supermix (Biorad, 1,708,882) on a Biorad CFX96 instrument with the following program: initial denaturation at 95 °C for 360 s; 40 cycles of 95 °C for 30 s, 55 °C for 60 s, and a melt curve step. The quantification cycle for the mRNAs of interest was normalized to TATA-box binding protein (TBP), hypoxanthine phosphoribosyltransferase (HPRT), or glyceraldehyde-3-phosphate dehydrogenase (GAPDH) reference mRNA, and data were expressed as fold change or log2 fold change over vehicle treatment. Gene-specific primer sequences are listed in Supplementary Table 3.

### Immunocytochemistry, image acquisition, and analysis

iPSC-derived neurons and primary cortical neuron cultures were fixed for 20 min with 4% paraformaldehyde (Thermo Fisher Scientific, 28,908) at RT. Three gentle washes with PBS (Thermo Fisher Scientific, 10,010,049) were followed by cell membrane permeabilization using 0.25% Triton X-100 (Millipore Sigma, 9400) for 8 min at RT. Cells were incubated with a blocking solution containing 5% bovine serum albumin (BSA) and 10% Normal Donkey Serum for 45 min at RT. The blocking solution was removed, and cells were incubated with primary antibodies diluted in fresh blocking solution overnight at 4 °C. Primary antibodies included anti-TUJ1 (1/250, Aves Lab, TUJ), anti-CTIP2 (1/100, abcam, ab18465), anti-p-IRF3 (1/50, Cell Signaling Technology, 29047S), anti-p-NF-κB (1/50, Santa Cruz Biotechnology, sc-136548), anti-phospho-histone H2A.X (γH2AX, 1/50, Cell Signaling Technology, 9718 T), anti-calnexin (1/50, Novus Biological, NB300-518), anti-STING (1/100, Proteintech, 19,851–1-AP; mouse cells), and anti-STING (1/50, R&D systems, AF6516; human cells). Antibodies are listed in Supplementary Table 2. After three washes with PBS, cells were incubated with species-specific Alexa Fluor (− 488, − 647, − 568; Thermo Fisher Scientific) secondary antibodies at a 1/500 dilution for 1 h at RT. Finally, the cells were incubated for 5 min with DAPI solution for nuclear staining and washed three times in PBS. Confocal images were acquired with an Image X-Press Micro Confocal (Molecular Devices) or LSM900 confocal microscope (Zeiss). Four z-stacks were acquired per field (1.6 μm step size), 15 to 25 fields per well using a 40 × objective for iPSC-derived neurons and a 20 × objective for primary mouse cortical neurons. Within each experiment, all groups were imaged with the same acquisition settings. Automatic quantifications were performed using a custom Fiji/ImageJ-based plugin (National Institutes of Health, version 1.53c) [86] for all cellular analyses except TDP-43 depletion, which was performed by blinded manual quantifications of γH2AX and STING-positive neurons performed on 80 to 100 cells per well. For area and integrated intensity measurements of stained cytoplasmic STING and nuclear p-IRF3 and p-NF-kB, and γH2AX, human iPSC-neurons and primary mouse cortical neurons were analyzed using a custom Fiji/ImageJ-based plugin (National Institutes of Health). To investigate ER-activated STING, the nuclear mask was dilated (10 iterations), and the perinuclear stained STING area and integrated intensity were analyzed within the dilated nuclear mask.

### Quantification and statistical analysis

All data were plotted and statistically analyzed on R-studio software (version 4.2.1). Boxplots and bars represented the mean and standard error of the mean. Unpaired two-tailed Student’s *t* tests (R-studio) were used to compare between groups. Differences were considered statistically significant for *p* values < 0.05. *p* values were depicted in graphs as *, **, ***, **** representing *p* < 0.05, *p* < 0.01, *p* < 0.001, and *p* < 0.0001, respectively.

### Data and code availability

Custom scripts used to analyze immunofluorescence and RNA-seq of published datasets are available at https://github.com/waingerlab/STING. Any additional information reported in this paper will be shared by the lead contact upon request.

## Results

### Unbiased analysis of expression datasets suggests neuronal activation of the STING pathway in ALS

We first wanted to investigate whether STING signaling may be present in central nervous system neurons and contribute to neurodegenerative disease, beyond its well-recognized role in immune cells [6]. We selected three publicly available high-quality transcriptional datasets of spinal motor neurons (SMNs): pure populations of SMNs derived from human iPSCs containing pathologic C9orf72 repeat expansions and isogenic control lines [1] or other fALS mutations (*TARDBP*^*G298S/*+^, *PFN1*^*G118V/*+^, and *SOD1*^*G85R/*+^) and isogenic control lines [35], as well as laser-captured postmortem SMNs from sALS cases and controls [49]. We analyzed the enrichment of 25 core genes of the cGAS–STING pathway, including 12 from a prior study [62] and 13 from the literature [52, 62, 68]. Gene set enrichment analysis (GSEA) demonstrated differential activation of the STING pathway in both ALS iPSC-derived SMN datasets, despite markedly different techniques for generating the SMNs from iPSCs, and the postmortem laser-captured ALS SMNs (Supplementary Fig. 1a). In contrast, a similar analysis using 30 randomly selected genes (the same number of genes analyzed in the STING pathway) did not show significant enrichment in ALS versus control samples for any dataset (Supplementary Fig. 1b). Compared to the well-controlled iPSC SMN datasets, the higher variability observed in the sALS dataset may reflect greater mechanistic heterogeneity present in postmortem sALS tissue as well as greater impact of background variation [94].

Using the same datasets, we then examined the downstream effectors of STING. Cytosolic double-stranded DNA preferentially activates the canonical STING–TBK1–IRF3 pathway [11]. In contrast, nuclear DNA damage can elicit the non-canonical STING pathway, which does not require the presence of cytoplasmic DNA and culminates in strong NF-κB activation [20, 39]. Synergistic STING amplification via these two pathways may occur [95] and induce an exacerbated neuroinflammatory response [30]. While selective activation of the canonical IRF3 signaling was previously reported in myeloid cells from C9orf72 patients [62], our unbiased analysis of vulnerable motor neurons from the three RNA-seq datasets showed enrichment of genes from both the canonical (IRF3) and non-canonical (NF-κB) pathways (Supplementary Fig. 1c). Consistent with the activation of STING by both canonical and non-canonical pathways, GSEA also highlighted an enrichment of genes related to DNA damage response (Supplementary Fig. 1d). Thus, we hypothesized that this dual pathway activation of both IRF3 and NF-κB components may contribute to the role of STING in neurons and their vulnerability to neurodegenerative diseases.

### Human postmortem fALS and sALS brains and spinal cords show STING activation in vulnerable ALS neurons

**Fig. 1:**
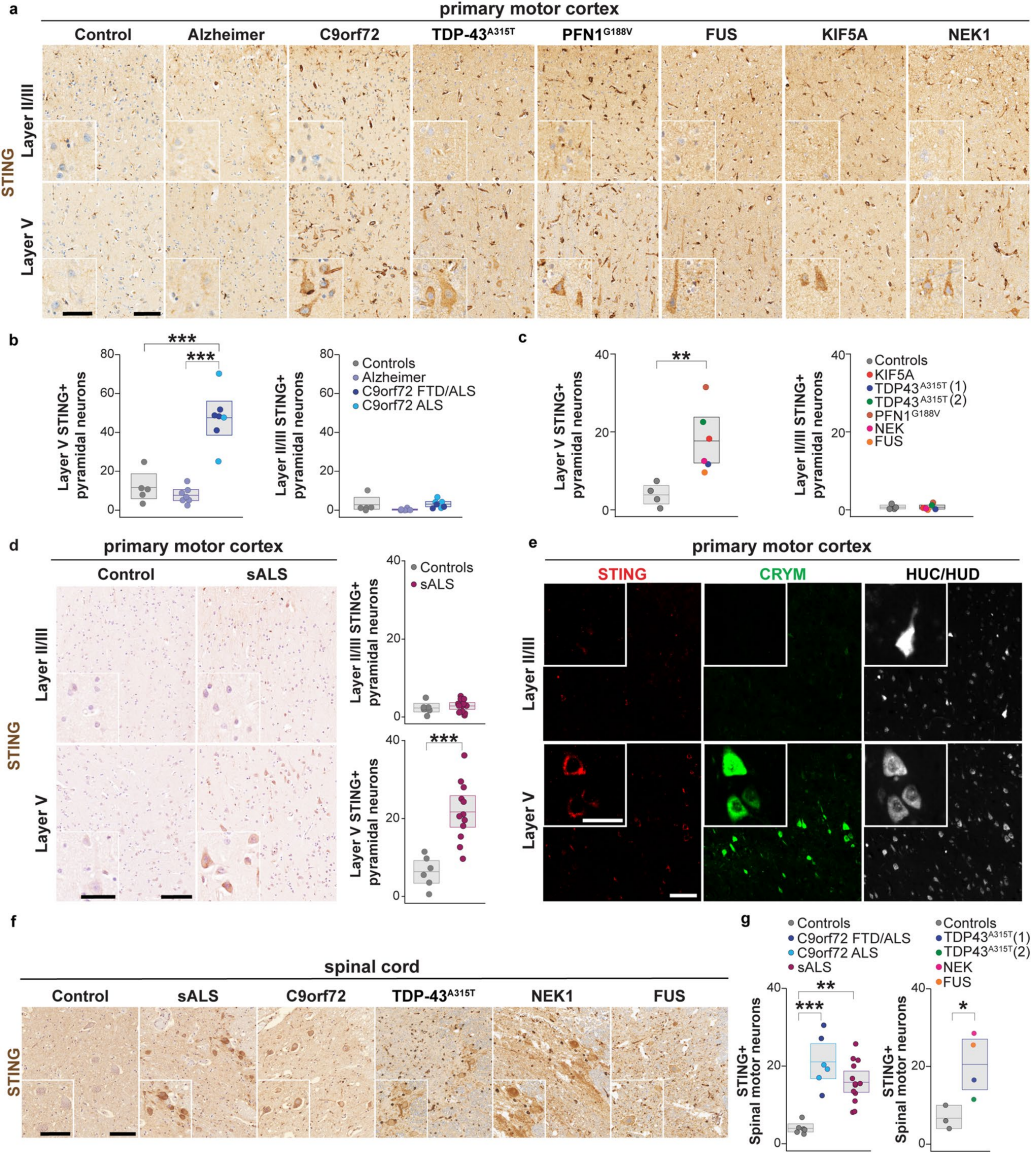
STING accumulates in layer V CRYM-positive cortical pyramidal neurons in the postmortem motor cortex and SMNs from multiple fALS and sALS variants. **a** Immunoperoxidase staining showing STING protein in layer II/III (upper panels) and layer V (lower panels) pyramidal neurons from matched non-neurological control, AD, C9orf72, as well as several additional fALS (mutations in TDP-43, PFN1, FUS, KIF5A*,* and NEK1) motor cortex brain sections. Bottom left inset in each picture shows high-magnification view of STING signal including in cell body and apical dendrite of layer V pyramidal neurons (bottom row). **b**, **c** Boxplots depicting the number of layer II/III and layer V STING-positive cortical pyramidal neurons in the motor cortex of C9orf72 (3 FTD/ALS dark blue dots and 5 ALS light blue dots) compared to AD (*n* = 8, purple) and matched non-neurological control (*n* = 5, gray) brains (**b**) and other fALS mutations (*n* = 6, colored as indicated) compared to separate additional matched non-neurological control brains (*n* = 4, gray) (**c**). Each dot represents an individual. **d** Left, immunoperoxidase staining showing STING protein in layer II/III and layer V pyramidal neurons from the motor cortex of sALS and separate additional matched non-neurological control brains. Right, boxplots depicting the number of layer II/III and layer V STING-positive cortical pyramidal neurons in the motor cortex of sALS (*n* = 12, purple) compared to non-neurological control (*n* = 6, gray) brains. **(e)** Representative immunofluorescence images from C9orf72 brains for neurons (HUC/HUD, white), CRYM (green), and STING (red). **f** Immunoperoxidase staining showing STING protein in ventral spinal cord SMNs from C9orf72*,* sALS, and additional fALS cases (TDP-43, FUS, NEK1) and matched non-neurological controls. **g** Boxplots depicting the number of STING-positive SMNs in C9orf72 (three FTD/ALS dark blue dots and three ALS light blue dots) and sALS (*n* = 12, purple) compared to matched non-neurological controls (*n* = 6, gray) and other fALS mutations (*n* = 4, colored as indicated) compared to separate additional matched non-neurological controls (*n* = 3). Scale bars = 100 μm and 40 μm for insets. All data are shown as mean $\pm$ s.e.m (boxplots), unpaired two-tailed Student’s *t* test: *p* < 0.05*; *p* < 0.01 **; *p* < 0.001 ***

To assess STING activation and its specificity for disease-relevant neurons, we first obtained postmortem motor cortex tissue sections from eight C9orf72 repeat expansion with ALS (five ALS alone and three ALS/FTD), eight phospho-TDP-43 (p-TDP-43)-negative Alzheimer’s disease (AD), and six non-neurological disease control brains. The tissues were matched for age, sex, and postmortem interval (Supplementary Table 1). The specificity of the STING antibody was validated using colon and spleen sections (high-level expression of STING) from wild-type (WT) versus STING goldenticket (gt) mice (STING^gt/gt^), which do not produce the protein [85](Supplementary Fig. 2a). Using 3,3’-diaminobenzidine (DAB) immunohistochemistry for STING, we identified only limited staining, which appeared to be restricted to vessels, in control sections (Fig. 1a). Glial STING activation was observed in AD and ALS cases (Supplementary Fig. 2b, left panels); cellular identities were confirmed by immunostaining for microglia (IBA1) and astrocytes (GFAP) (Supplementary Fig. 2b, right panels). Layer V cortical motor neurons, Betz cells in humans, are among the most affected neurons in ALS [32, 72, 84]. In contrast to AD and non-neurological disease control brains, all C9orf72 ALS brains examined showed punctate signal within the neurons of typical layer V Betz morphology (Fig. 1a, lower panels), including large cell body and apical dendrite. Quantification showed a five-fold increase in layer V STING-positive neurons in C9orf72 sections compared to AD and non-neurological disease controls (Fig. 1b, left panel), whereas no increase was observed in the superficial layer II/III neurons (Fig. 1b, right panel). We found similar results in the brains of individuals with other fALS gene mutations, including TDP-43, PFN1, FUS, KIF5a, and NEK1, again with increased STING signal in deep layer V pyramidal neurons but not layer II/III neurons (Fig. 1a, c).

To determine whether STING activation was also observed in sALS, we measured STING levels in postmortem motor cortex tissue sections from 12 sALS and 6 non-neurological disease-matched control brains. We observed a markedly increased number of STING-positive layer V neurons in sALS compared to control sections, with few STING-positive superficial layer II/III neurons in sALS or control sections (Fig. 1d).

To confirm our ability to identify layer V pyramidal cortical neurons based on DAB staining morphology and to show definitively that the increase in STING expression in the C9orf72 brains was neuronal and specific for layer V neurons, we took advantage of the mu-crystallin (CRYM) marker, which labels layer V cortical motor neurons [4, 66] and detects their degeneration in ALS mouse models [61]. Staining for CRYM protein in the human motor cortex sections showed selectivity for deep layer V pyramidal neurons (Supplementary Fig. 2c). Co-staining with the neuronal marker HUC/HUD, CRYM, and STING confirmed the presence of increased neuronal STING activation within layer V cortical motor neurons in C9orf72 ALS brains (Fig. 1e). Consistent with the DAB results, the activation of STING had a remarkable layer V specificity, as STING activation was strong in layer V but nearly absent in layers II/III (Fig. 1e, upper vs lower). Within the STING-positive layer V neurons, we also detected activation of the downstream effector p-IRF3 in the nucleus of CRYM-positive neurons (Supplementary Fig. 2d).

We next evaluated whether STING activation may also occur in SMNs, the other focus of greatest ALS vulnerability in addition to layer V cortical motor neurons. Spinal cord samples obtained from individuals with fALS and sALS revealed punctate signals within the SMNs of the ventral horn (Fig. 1f, left panel) compared to non-neurological disease controls. A quantitative analysis demonstrated a four-fold increase in STING-positive SMNs in the ventral horn of sALS cases (*n* = 13), a five-fold increase in fALS cases with *C9orf72* mutation (*n* = 6), and a three-fold increase in fALS cases with other mutations (*n* = 4), compared to non-neurological disease controls (Fig. 1g, right panel).

To investigate whether neuronal STING activation is limited to the most disease-relevant regions, we analyzed a brain region largely spared in ALS, the occipital cortex, from sALS and C9orf72 cases compared to non-neurological disease controls. In contrast to our results in the primary motor cortex, no sALS or C9orf72 ALS brains showed STING activation in either layer V or layer II/III neurons (Supplementary Fig. 2e). These findings collectively indicate neuronal activation of the STING pathway specifically in vulnerable cortical motor neurons and SMNs of sALS and a broad range of fALS cases.

### STING signaling is increased in a *C9orf72* mouse model

**Fig. 2:**
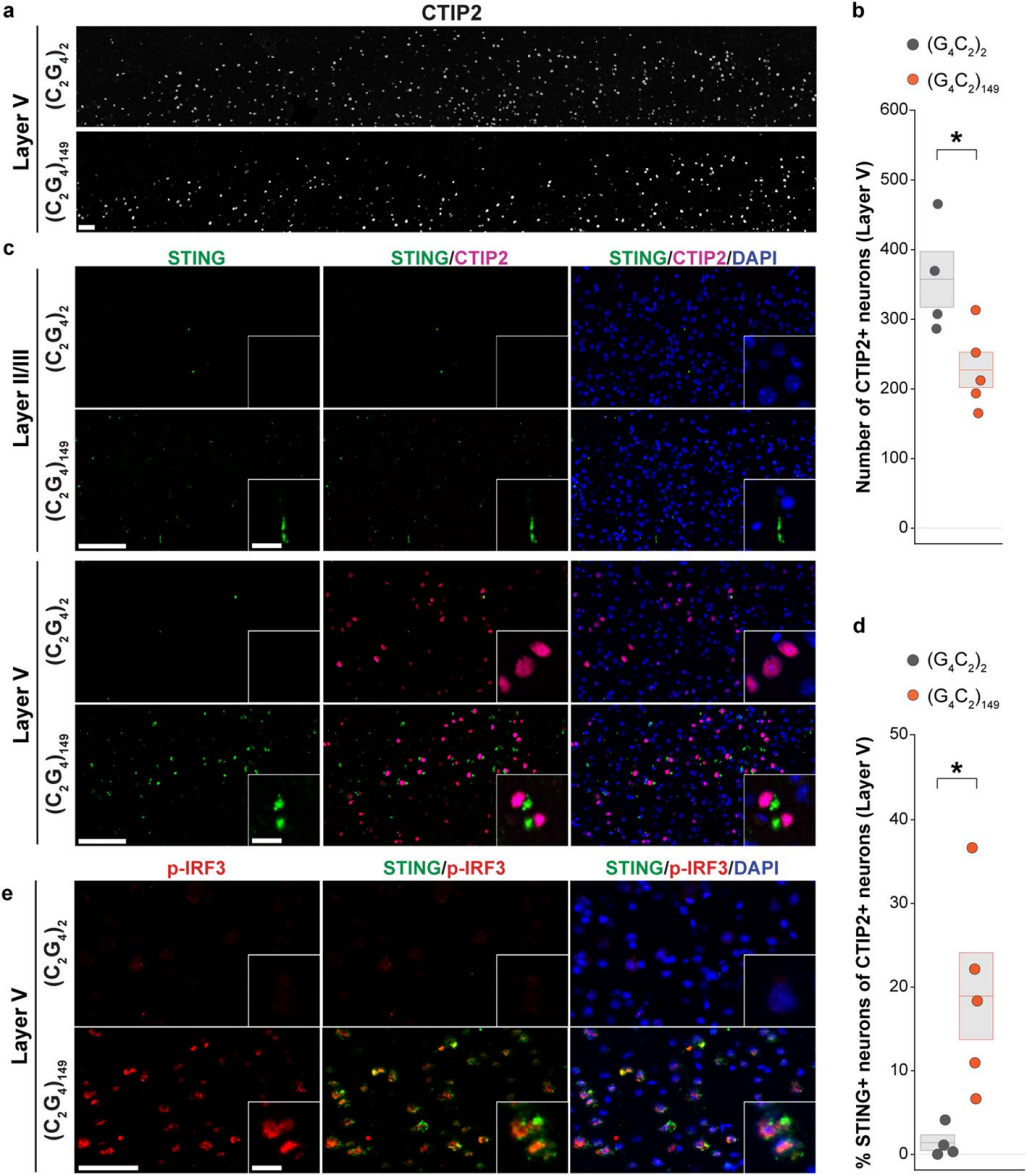
STING signaling is increased in deep layer V neurons in *C9orf72* mouse model. **a** Representative images of layer V CTIP2-positive cortical pyramidal neurons (white) in the motor cortex of 1-year-old mice expressing 149 G_4_C_2_-repeats ((G_4_C_2_)_149_, three males, two females) compared to 2 G_4_C_2_-repeats control ((G_4_C_2_)_2_, two males, two females). **b** Boxplot depicting the number of layer V CTIP2-positive neurons in (G_4_C_2_)_2_ (gray) and (G_4_C_2_)_149_ (orange) mice. **c** Immunostaining for STING (green), CTIP2 (magenta), and DAPI (blue) in layer II/III (above) and layer V (below) in the motor cortex of (G_4_C_2_)_2_ control and (G_4_C_2_)_149_ mice. Representative images for layer II/III and layer V are from the same motor cortex tissue sections. Bottom right inset in each image shows high-magnification view. **d** Boxplot represents the percentage (%) of layer V CTIP2-positive neurons that were also STING-positive (dual CTIP2+, STING+) in (G_4_C_2_)_149_ mice compared (G_4_C_2_)_2_ to mice. **e** Representative images showing cytoplasmic STING (green) and adjacent downstream marker p-IRF3 (red) in the nucleus. Each dot in boxplots represents a mouse (*n* = 4–5 per group). Scale bars = 50 μm and 10 μm for insets. All data are shown as mean $\pm$ s.e.m, unpaired two-tailed Student’s *t* test: *p* < 0.05*; *p* < 0.01 **; *p* < 0.001 ***

To test whether the observed increase of STING activation in postmortem tissue may also be present during earlier stages of ALS in vivo, we examined motor cortex sections from mice injected with AAVs expressing either 2 or 149 repeats of (G_4_C_2_) (AAV (G_4_C_2_)_2_ or AAV (G_4_C_2_)_149_, respectively), an established model of C9orf72 repeat-associated disease pathology [13, 16]. For mouse studies, we identified layer V cortical motor neurons using the COUP-TF-interacting protein 2 (CTIP2), a nuclear marker that specifically labels layer V neurons [4, 66], including cortical motor neurons [23, 61]. Indeed, when we first quantified CTIP2-positive cortical motor neurons in motor cortices of 1-year-old animals, we found a significant reduction (36.4%) in AAV (G_4_C_2_)_149_ compared to control AAV (G_4_C_2_)_2_ motor cortices (Fig. 2a, b). In contrast, the number of BRN2-positive layer II/III neurons was unchanged between AAV (G_4_C_2_)_149_ and AAV (G_4_C_2_)_2_ motor cortices (Supplementary Fig. 3a).

We then investigated whether STING was activated in motor cortices of AAV (G_4_C_2_)_149_ mice and whether this activation occurred selectively in vulnerable deep layer V cortical motor neurons. We did not detect neuronal STING accumulation in either AAV (G_4_C_2_)_149_ or AAV (G_4_C_2_)_2_ brains in superficial cortical layers (Fig. 2c upper panels). However, co-immunostaining of STING with CTIP2 revealed a significant accumulation of STING within layer V neurons of AAV (G_4_C_2_)_149_ but not AAV (G_4_C_2_)_2_ sections (Fig. 2c; lower panels of Fig. 2d, Supplementary Fig. 3b). We observed increased STING staining immediately adjacent to CTIP2-positive nuclei (18.9% of CTIP2 + neurons), consistent with the typical perinuclear spatial pattern of activated STING and the results in the human postmortem tissue (Fig. 2d). Consistent with the differential STING activation, p-IRF3 was increased in AAV (G_4_C_2_)_149_ mice but not in AAV (G_4_C_2_)_2_ control mice. Furthermore, within the AAV (G_4_C_2_)_149_ cortices, p-IRF3 was restricted to deep layers and almost exclusively co-localized within STING-positive neurons (Fig. 2e). Increased p-NF-κB was also observed in the cortices of AAV (G_4_C_2_)_149_ compared to AAV (G_4_C_2_)_2_ animals (Supplementary Fig. 3c). Thus, the mouse model studies confirmed the specificity for vulnerable neurons revealed in the human postmortem tissue: STING activation in the motor cortex occurred specifically within susceptible layer V CTIP2-positive cortical motor neurons of (G_4_C_2_)_149_ but not (G_4_C_2_)_2_ mice.

### The STING pathway is present and functional in primary mouse cortical neurons and healthy control human iPSC-derived neurons

**Fig. 3:**
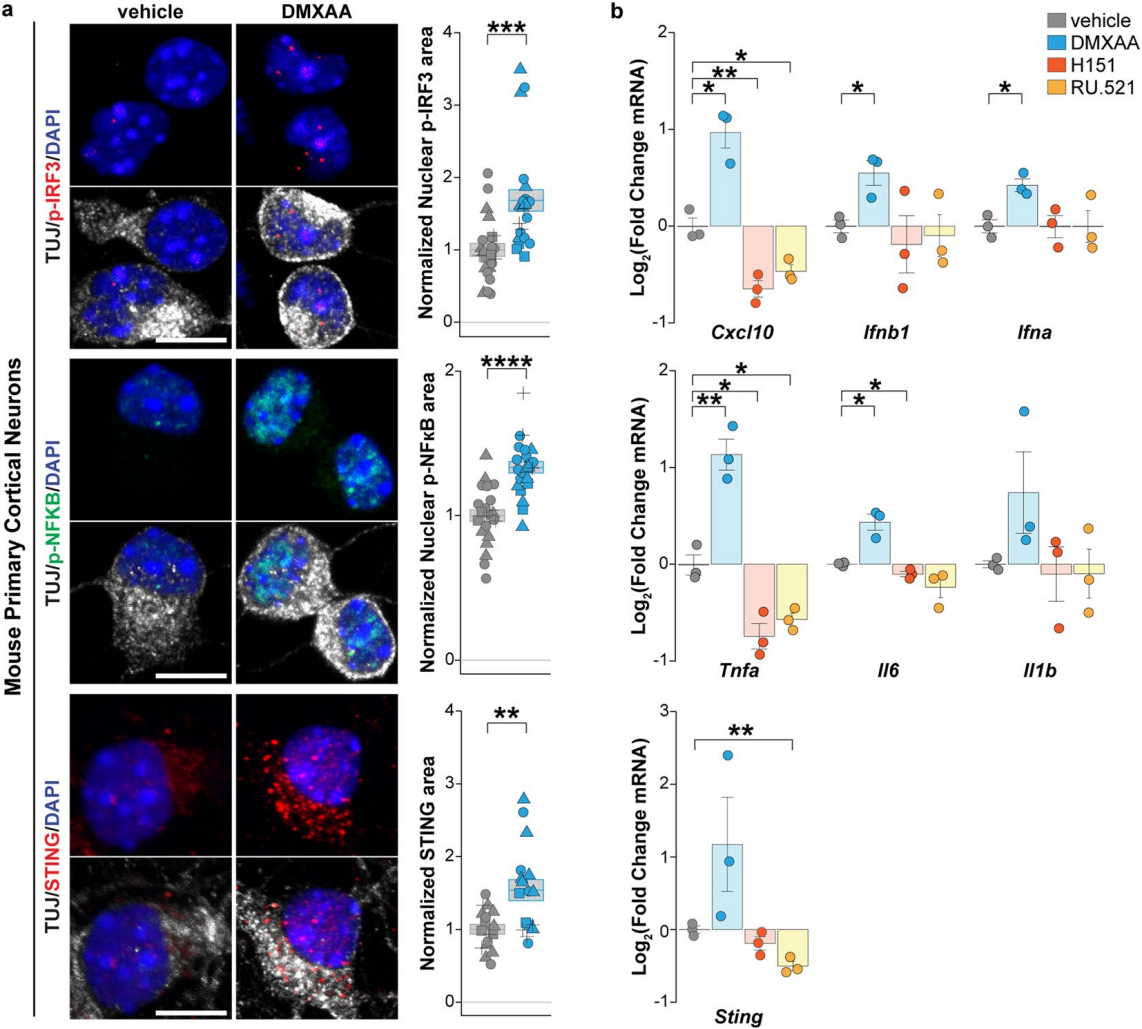
STING pathway is present and functional within primary mouse cortical neurons. **a** Left, immunofluorescence staining of canonical p-IRF3 and non-canonical p-NF-κB effectors and their upstream regulator STING protein in primary mouse cortical neurons treated with mouse STING agonist DMXAA (20 μg/ml, 1 h) compared to vehicle control. Neuron-specific class III β-tubulin (TUJ1, white) and DAPI staining (blue) demarcate neurons and cell nuclei, respectively. Right, quantification of nuclear p-IRF3 (upper panel), nuclear p-NF-κB (middle panel), and cytoplasmic STING areas in primary mouse cortical neurons treated with vehicle control (gray) and DMXAA (blue). Each object represents a well, and each symbol represents an independent differentiation (triangles, squares, circles, and crosses). **b** RT-qPCR analysis of the RNA expression of canonical IRF3 (above), non-canonical NF-κB response cytokines (middle), and STING (below) in primary mouse cortical neurons treated (3 h) with vehicle control (gray), DMXAA (blue, 20 μg/ml), or with STING blockers H151 (red, 1 μM) and RU.521 (orange, 10 μM). Each dot represents an independent experiment (*n* = 3). Scale bar = 10 μm. Data are shown as mean $\pm$ s.e.m, unpaired two-tailed Student’s *t* test: *p* < 0.05*; *p* < 0.01 **; *p* < 0.001 ***

STING signaling has been well characterized in microglia and immune cells [11, 41, 74] but has not been previously documented in central nervous system neurons. To interrogate the functional capacity of STING signaling in neurons, we treated primary mouse cortical neurons and cortical-like neurons derived from human healthy control iPSCs via NGN2 overexpression [104] with modulators of the STING pathway (Fig. 3, primary mouse neurons; Supplementary Fig. 4, iPSC-derived neurons). Treating neurons with a STING agonist (DMXAA and cGAMP for mouse and human neurons, respectively) increased STING pathway activation, indicated by phosphorylation of the downstream nuclear regulators IRF3 (p-IRF3) and NF-κB (p-NF-κB) (Fig. 3a upper and middle panels) and increased expression of canonical IRF3 (*Cxcl10*,* Ifnb1*,* Ifna*) and non-canonical NF-κB (*Il6*,* Il1b*,* Tnfa*) downstream target inflammatory markers as measured by RT-qPCR (Fig. 3b upper and middle panels; Supplementary Fig. 4a) [2, 15, 20, 38, 90]. Next, we assessed the effects of inhibiting any potential basal STING activity using inhibitors of STING [31] and cGAS, H-151 [31] and RU.521 [96], respectively. These inhibitors yielded reduction in the intrinsic levels of the downstream cytokines *Tnfa*, *Il6*, and *Cxcl10*, indicating the presence of some baseline level of STING signaling within the neurons (Fig. 3b). Notably, we also observed modulation of the STING protein and RNA, which were both increased by STING agonists and decreased by STING inhibitors, consistent with literature documenting a positive feedback loop on STING transcription [33, 58] (Fig. 3a, b lower panels; Supplementary Fig. 4b). Thus, both STING and its established regulatory and downstream signaling pathways are present, functional, and modifiable within central nervous system neurons.

### SMNs and NGN2 neurons derived from a range of human fALS iPSCs show neuron-autonomous STING signaling activation

**Fig. 4:**
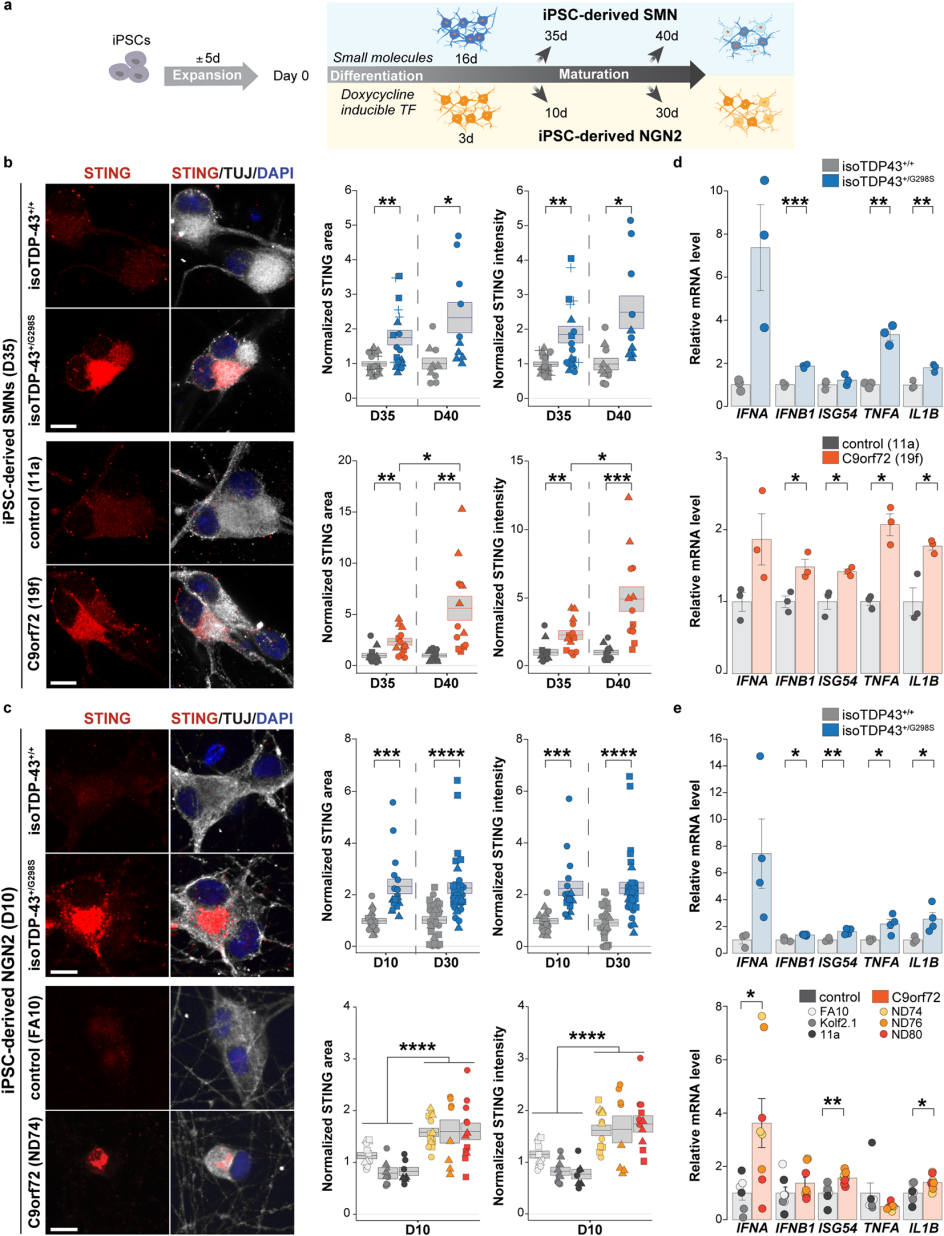
STING pathway is activated in a range of human ALS iPSC-derived neurons in the absence of exogenous stressors.** a** Schematic for iPSC-derived small-molecule SMN differentiation and NGN2 transcription factor (TF)-based induction. **b** Left, immunofluorescence staining for STING (red), TUJ1 (white), and DAPI (blue) in iPSC-derived small-molecule SMNs at day 35 of differentiation (D35) from isoTDP-43^+/+^ control compared to isoTDP-43^+/G298S^ (upper panels) and healthy control 11a compared to *C9orf72* 19f SMNs (lower panels). Right, quantification of time-dependent changes in cytoplasmic STING area and STING intensity in isoTDP-43^+/+^ (gray) compared to isoTDP-43^+/G298S^ (blue) (above) and healthy control (11a, dark gray) compared to C9orf72 (19f, orange) (below) SMNs. **c** Left, staining for STING (red), TUJ1 (white), and DAPI (blue) in iPSC-derived TF NGN2 neurons at day 10 of differentiation (D10) from isoTDP-43^+/+^ compared to isoTDP-43^+/G298S^ (upper panels) and healthy control (FA10) compared to C9orf72 (ND74) (lower panels) NGN2 neurons. Right, quantification of time-dependent changes in cytoplasmic STING area and STING intensity in isoTDP-43^+/+^ (gray) compared to isoTDP-43^+/G298S^ (blue) (above) and healthy controls (gradient of gray: light, FA10, medium, Kolf2.1; dark, 11a) compared to C9orf72 (yellow, ND74; orange, ND76; red, ND80) (below) NGN2 neurons. **d** RT-qPCR analysis showing increase of canonical IRF3 (*IFNA*, *IFNB1*, *ISG54*) and non-canonical NF-κB (*TNFA*, *IL1B* response genes in isoTDP-43^+/+^ (gray) compared to isoTDP-43^+/G298S^ (blue) (above) and healthy control (11a, dark gray) compared to C9orf72 (19f, orange) (below) SMNs at day 35. **e** RT-qPCR analysis of downstream canonical IRF3 and non-canonical NF-κB response genes in isoTDP-43^+/+^ (gray) compared to isoTDP-43^+/G298S^ (blue) at day 30 (above) and healthy control (gray, same three indicated lines) compared to C9orf72 (orange, same three indicated lines) (below) NGN2 neurons at day 10. **b**, **c** Each object represents a well, and each symbol represents an independent differentiation (triangles, squares, and circles). **d**,** e** Each dot represents an independent differentiation (n = 3–4). Scale bar = 10 μm. All data are shown as mean $\pm$ s.e.m, unpaired two-tailed Student’s *t* test: *p* < 0.05*; *p* < 0.01 **; *p* < 0.001 ***; *p* < 0.0001 ****

To determine whether STING is activated in ALS iPSC-derived neurons without the influence of other cell types, we analyzed levels of STING and its downstream mediators in SMNs and NGN2 neurons derived from multiple ALS and control iPSC lines (Fig. 4a), including an isogenic pair of TDP-43 lines (isogenic unedited control (isoTDP-43^+/+^) and an edited line into which an fALS mutation was introduced (isoTDP-43^+/G298S^)) (Fig. 4) [77], an independent TDP-43^+/G298S^ ALS subject line (TDP-43^+/G298S^) (Supplementary Fig. 5a), a range of C9orf72 repeat expansion lines, and separate control lines (Fig. 4). In both iPSC-derived SMNs and NGN2 neurons, we observed a time-dependent increase in STING activation within ALS compared to control neurons (Fig. 4b, c; Supplementary Fig. 5a) and a parallel increased expression level of both canonical and non-canonical downstream genes (Fig. 4d, e; Supplementary Fig. 5b,c), together suggesting a hyperactive innate immune response in these ALS neurons. Thus, STING activation in ALS iPSC neurons occurs in a neuron-autonomous and time-dependent manner. It begins after only brief culture periods, does not require additional stressors, and includes both transcriptional and translational components.

Given that STING is present within and adjacent to the endoplasmic reticulum and has a predominant perinuclear localization when activated [18, 27, 29], we next characterized the subcellular location of STING within ALS neurons [40]. STING partially co-localized with the ER marker calnexin but also included substantial perinuclear staining (Supplementary Fig. 5d). To quantify the perinuclear component, we measured STING levels adjacent to the nucleus by dilating a mask generated from the nuclear DAPI signal and observed increased amounts in ALS compared to control neurons (Supplementary Fig. 5e).

### Activation of STING in neurons by DNA damage

**Fig. 5:**
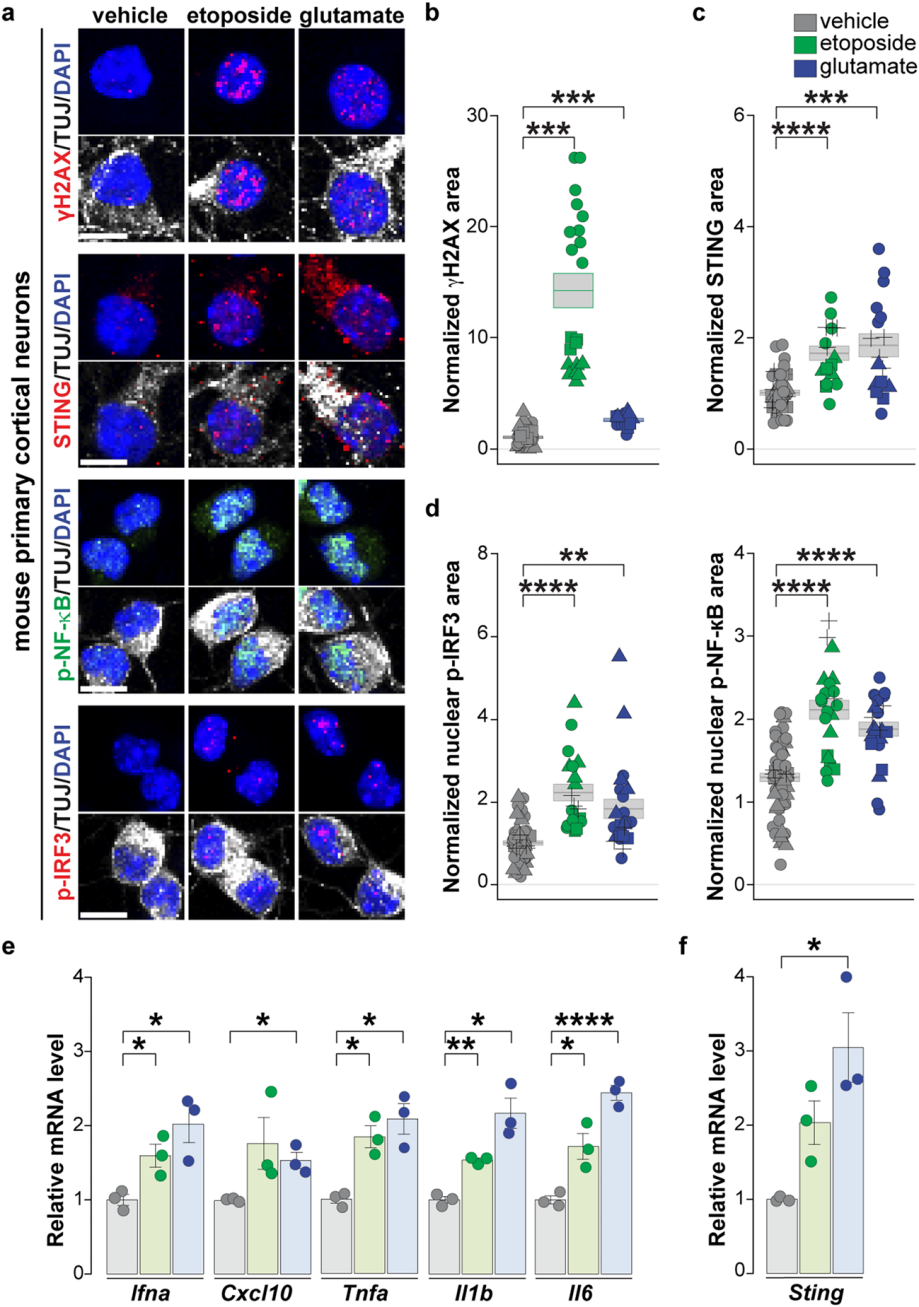
DNA damage induction upon etoposide or glutamate treatment yields γH2AX and STING pathway activation in primary mouse cortical neurons. **a** Representative immunofluorescence staining of γH2AX (red), STING protein (red) and downstream effectors, p-IRF3 (red), and p-NF-κB (green) in primary mouse cortical neurons after 1 h treatment with either vehicle, DNA damage stressors etoposide (5 μM) or glutamate (10 μM). Cell nuclei and neuronal cytoplasm are demarcated by DAPI (blue) and TUJ1 staining (white), respectively. **b-d** Quantification of nuclear γH2AX area (**b**), cytoplasmic STING area (**c**), and downstream effectors, nuclear p-IRF3 (**d**, left) and p-NF-κB (**d**, right). **e** RT-qPCR analysis of downstream inflammatory-response genes following vehicle (gray), etoposide (green), and glutamate (blue) after 3 h treatments as above. **f** RT-qPCR analysis of *Sting* expression in response to vehicle (gray), etoposide (green), and glutamate (blue) treatment (3 h). **b, c, d** Each object represents a well, and each symbol represents an independent experiment (triangles, squares, circles, and crosses). **e, f** Each dot represents an independent experiment (n = 3). Scale bar = 10 μm. All data are shown as mean $\pm$ s.e.m, unpaired two-tailed Student’s *t* test: *p* < 0.05*; *p* < 0.01 **; *p* < 0.001 ***; *p* < 0.0001 ****

A primary role of STING is to activate an innate immune response to DNA damage, and neuronal DNA damage accumulation is a key early feature [88] and a potential mechanistic driver in neurodegenerative diseases, including AD, ALS, and FTD [14, 21, 47, 60, 69, 78]. To investigate whether DNA damage elicits STING activation in neurons, we treated primary mouse cortical neurons with etoposide, which damages nuclear DNA by inhibiting type II topoisomerase [101], and observed both increased nuclear γH2AX, a marker of nuclear DNA damage and repair (Fig. 5a,b), and cytoplasmic STING levels (Fig. 5a,c). Increased nuclear translocation of both p-IRF3 and p-NF-κB and elevated downstream inflammatory gene expression indicated activation of both canonical and non-canonical STING pathways (Fig. 5a,d,e).

One potential mechanism of DNA damage in ALS relates to cortical hyperexcitability, which occurs in both ALS and FTD [8, 63, 97], and may be related to increased glutamatergic signaling [83]. Notably, glutamate receptor agonists can cause DNA damage in parallel with increased neuronal activity [59, 91]. We extended this reasoning to investigate whether DNA damage resulting from increased neuronal excitability would also induce STING pathway activation. Short pulses of glutamate (1 h) yielded increases in nuclear γH2AX and cytoplasmic STING levels (Fig. 5a–c). STING was elevated at both the protein and RNA levels (Fig. 5c, f). We again observed increased levels of both nuclear p-IRF3 and p-NF-κB and their respective downstream target genes by RT-qPCR (Fig. 5d, e).

### Both TDP-43 depletion and C9orf72 dipeptide repeat proteins lead to DNA damage and STING pathway activation in iPSC-derived neurons

**Fig. 6:**
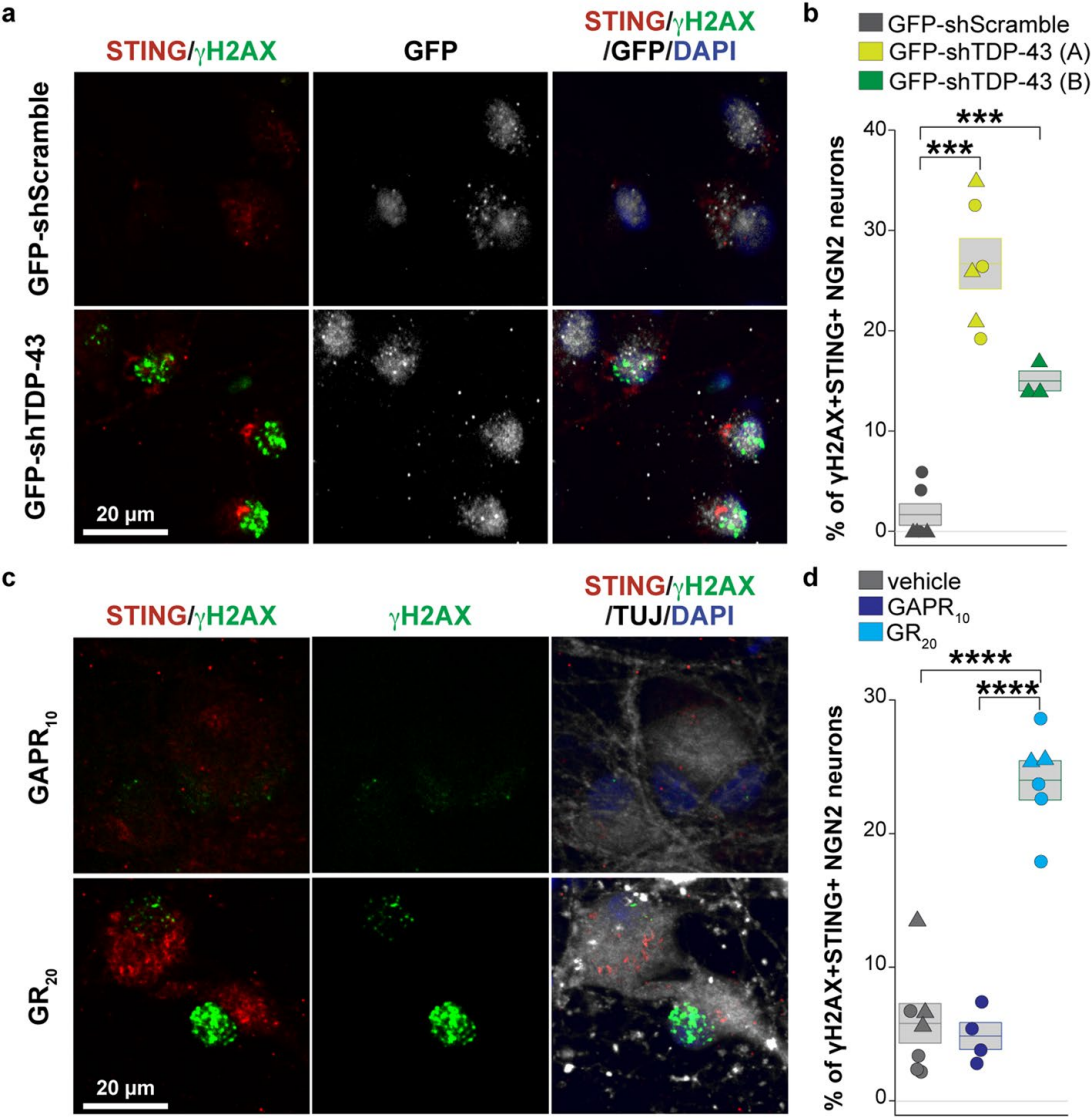
TDP-43 depletion and C9orf72 DPR treatment each elicits DNA damage and STING pathway activation in iPSC-derived neurons. **a** Representative immunofluorescence images of DNA damage (γH2AX, green) and STING (red) in control iPSC-derived NGN2 neurons treated with GFP-shRNA-TDP-43 for 48 h compared to GFP-shRNA-Scramble (GFP, white). **b** Quantification of dual DNA damage γH2AX-positive and STING-positive NGN2 neurons following treatment with either of two shRNA-TDP-43 constructs (yellow GFP-shTDP-43 (A); green GFP-shTDP-43 (B)) compared to scrambled shRNA (gray GFP-shScramble). Each object represents a well, and each symbol represents an independent experiment (circles, triangles). **c** Representative immunofluorescence images of γH2AX (green), STING protein (red), TUJ1 (white), and DAPI (blue) in control iPSC-derived NGN2 neurons after 24 h treatment with (GR)_20_ dipeptide repeat compared to (GAPR)_10_ dipeptide repeat control (1.25 μM). **d** Quantification of dual DNA damage γH2AX + and STING + NGN2 neurons following treatment with (GR)_20_ compared to control (GAPR)_10_ and an additional DMSO vehicle control. Each object represents a well, and each symbol represents an independent experiment (circles, triangles). Scale bar = 20 μm. All data are shown as mean $\pm$ s.e.m (boxplot **b**, **d**), unpaired two-tailed Student’s *t* test: *p* < 0.05*; *p* < 0.01 **; *p* < 0.001 ***, *p* < 0.0001 ****

Two additional ALS- and FTD-related processes that have been implicated in DNA damage include TDP-43 dysregulation [25, 28, 37, 48, 50, 64, 100, 106] and C9orf72 dipeptide repeat (DPR) production [3, 22, 56, 73, 98, 105]. We considered whether these features also elicit STING activation. First, we performed TDP-43 knockdown by using GFP-labeled lentiviral particles containing shRNA against *TARDBP* in control iPSC-derived NGN2 neurons and analyzed the number of STING- and γH2AX-positive neurons 48 h after transduction. ShRNA-mediated knockdown of TDP-43 via either of the two tested shRNA TDP-43 lentiviral particles resulted in significantly increased DNA damage and STING activation compared to a shRNA scramble control (Fig. 6a, b).

Next, we modeled C9orf2 DPR toxicity by applying exogenously synthesized GR_20_ [22] in iPSC-derived NGN2 neurons for 24 h and analyzed the number of STING and γH2AX positive neurons [67]. We found that GR_20_ dipeptide repeats caused not only DNA damage but also STING activation compared to either a control (GAPR)_10_ peptide [67] or vehicle control (Figs. 6c, d). These findings demonstrate that both TDP-43 depletion and exposure to GR dipeptides lead to DNA damage and STING activation in iPSC-derived NGN2 neurons. The results provide disease-relevant mechanisms that link DNA damage to STING and innate immune activation across different ALS variants.

### STING pathway inhibition abrogates the enhanced inflammatory signal in ALS iPSC-derived neurons

**Fig. 7:**
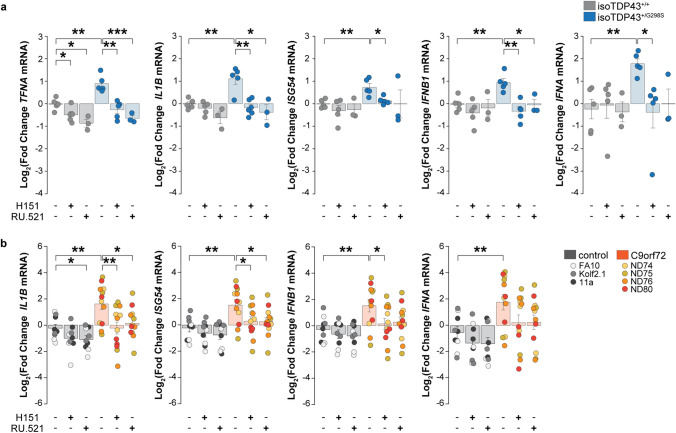
Enhanced inflammatory response in ALS iPSC-derived neurons is suppressed by STING pathway inhibition. RT-qPCR analysis of the expression of non-canonical NF-κB (*TNFA*, *IL1B*) and canonical IRF3 (*ISG54*, *IFNB1*, *IFNA*) target genes in (**a**) isoTDP-43^+/+^ control (gray) compared to isoTDP-43^+/G298S^ (blue) and in (**b**) healthy controls (gradient of gray: light, FA10, medium, Kolf2.1; dark, 11a) compared to C9orf72 (yellow, ND74; orange, ND76; red, ND80) NGN2 neurons treated for 24 h with vehicle control, or with STING blockers H151 (1 μM) and RU.521 (10 μM). Each dot represents an independent differentiation (*n* = 5 for TDP isogenic pair in (**a**), and n = 3 for C9orf72 and controls in (**b**)). All data are shown as mean $\pm$ s.e.m, unpaired two-tailed Student’s *t* test: *p* < 0.05*; *p* < 0.01 **; *p* < 0.001 ***

We have now identified potential disease-relevant activators of the STING pathway in ALS iPSC-derived neurons and demonstrated that such mechanisms are sufficient to yield STING activation and stimulation of downstream inflammatory cytokines. To show that STING is indeed necessary for the observed elevated innate immune signaling present in ALS iPSC-derived neurons, we treated cells with STING pathway blockers and assessed the effects on downstream cytokines. We observed that both the STING inhibitor H151 and the cGAS inhibitor RU.521 suppressed the increased interferon response gene expression for both canonical and non-canonical STING pathways in NGN2 neurons derived from isoTDP-43^+/G298S^ compared to isoTDP-43^+/+^ iPSC lines (Fig. 7a) and several C9orf72 repeat expansion compared to control iPSC lines (Fig. 7b). These findings establish the contribution of STING signaling to neuron-intrinsic inflammatory signaling in ALS.

## Discussion

While the timing, drivers, and consequences of neuroinflammation in neurodegenerative diseases remain contested, the prevailing conceptual framework involves an initial neuronal insult after which inflammatory cells initiate an innate immune response that leads to neuroinflammation and eventually neurodegeneration [79]. Here, we demonstrated that STING activation occurred within vulnerable neurons in ALS/FTD through a neuron-autonomous mechanism, and the activation included both the canonical IRF3 and non-canonical NF-κB pathways. We provided concordant evidence from human postmortem cortices and spinal cords across a broad range of fALS mutations and sALS, a mouse model of C9orf72 repeat expansion ALS/FTD, and iPSC-derived neurons harboring fALS mutations. Each of these approaches confirmed important features of the STING neuronal process in ALS/FTD: the human postmortem studies showed the relevance to both sALS and fALS and the specificity for the most disease-susceptible cell types, the layer V cortical motor neurons and SMNs; the *C9orf72* mouse model suggested that STING activation occurred at relatively early time periods in the disease course; finally, the iPSC modeling studies supported the neuron-autonomous capacity of the STING signaling process.

Intrinsic neuronal innate immune responses have been recently identified in neurons as potential contributors to Huntington’s disease and C9orf72 ALS [53, 82, 105], although the proposed mechanisms have been RNA rather than DNA-based. Among the pathogen-associated molecular pattern (PAMP) and damage-associated molecular pattern (DAMP) receptors available to produce host-initiated neuroinflammation [26, 51], the STING pathway appears to be well positioned to respond to DNA damage, which lies at the crossroads of pathophysiological mechanisms in ALS/FTD. DNA damage results from mutations in numerous fALS genes [71, 80, 99], C9orf72 repeat expansion [3, 22, 56, 73, 98, 105], hyperexcitability [59, 91], and nuclear TDP-43 depletion [25, 28, 48, 50, 64, 100], and we now extend those studies to demonstrate that DNA damage from each leads to STING activation in neurons. Beyond these mechanisms, de-repression of endogenous retroviruses [54] may serve as another source of DNA damage and STING activation [94]. In addition to nuclear DNA damage, STING may be activated by mitochondrial damage and subsequent mitochondrial DNA release [11, 17], a process that occurs following TDP-43 overexpression [102]. Other ALS pathological mechanisms may amplify STING activation. For example, reduced clearance of STING by autophagy inhibition can yield increased STING signaling, as shown after *C9orf72* depletion in myeloid cells [62]. Deficits of autophagy in ALS iPSC neuronal modeling studies share similar implications [7], although interpretation is complicated by the role of STING in promoting autophagy at least in some cell types [29]. Links between autophagy and the distinct mechanisms of nuclear DNA damage and DNA release from damaged mitochondria may form additional mechanistic layers [70, 81, 98].

In the ALS iPSC-derived neurons, upregulation of both STING and its downstream signaling pathways occurred at surprisingly early times––day 10 in NGN2 neurons and day 35 in small-molecule-derived SMNs. At such times, robust disease-relevant phenotypes have proved hard to detect [34]. Notably, the activation of STING and its downstream effectors occurred without the addition of pharmacological stressors, which have been necessary to model many of the core neuropathological features within iPSC-derived neurons [34]. In contrast with STING activation in myeloid cells following *C9orf72* depletion, activation included both the canonical IRF3 pathway and the non-canonical NF-κB pathway, the latter occurring in response to nuclear DNA damage without requiring cytoplasmic DNA release and detection [20]. Moreover, interactions driven by STING activation between neurons and neighboring glial cells may potentiate the intrinsic neuronal inflammatory response.

What determines the selective vulnerability of specific neuronal subtypes among different neurodegenerative diseases remains a central unanswered question. The use of cortical layer-specific markers, including CRYM and CTIP2 in human and mouse studies, respectively, allowed us to confirm increased STING activation in layer V neurons within the motor cortex. In contrast, such activation was absent in the superficial layer II/III neurons within the motor cortex and throughout the occipital cortex, both largely spared in ALS. Based on our findings of STING activation in postmortem ALS SMNs, we expected and obtained concordant results with iPSC-derived SMNs. We did not necessarily expect that iPSC-derived NGN2 neurons would exhibit the same phenotype, given that they have been described as general models of excitatory cortical neurons. However, these neurons do not map well onto the subtypes of *bona fide* human cortical neurons [55, 104], and their expression of deep layer markers, such as *FEZF2* and *PCP4* [35], may be sufficient to drive some deep-layer phenotypes, in this case, STING activation in the presence of fALS mutations. Further investigation will be necessary to determine the specific pathways that elicit STING activation and the connections to selective vulnerability in ALS/FTD.

The functional integrity of STING signaling, observed within both primary mouse and human iPSC-derived neurons, and the robust STING activation in ALS iPSC-derived neurons establish a neuron-autonomous aspect of STING signaling. Blockage of STING within such neurons abrogates the immunophenotype of increased interferon signaling observed in ALS iPSC-derived neurons. Understanding how STING activity within neurons contributes to the broader contexts of neuroinflammation and neurodegeneration will require cell-type selective modulation of STING and other implicated innate immune pathways [82, 105]. Through independent and joint modulation of these pathways in co-culture and mouse models, novel aspects of these pathways in neurons may be dissected from their more traditional roles within microglia and astrocytes.

### Supplementary Information

Below is the link to the electronic supplementary material.

Supplementary file1 (PDF 10990 KB)

## Data Availability

Custom scripts used to analyze immunofluorescence and existing RNA-seq of published datasets are available at https://github.com/waingerlab/STING. Additional information will be shared by the corresponding author upon reasonable request.

## References

[1] Abo-Rady M, Kalmbach N, Pal A, Schludi C, Janosch A, Richter T, Freitag P, Bickle M, Kahlert AK, Petri S (2020). Knocking out C9ORF72 Exacerbates Axonal Trafficking Defects Associated with Hexanucleotide Repeat Expansion and Reduces Levels of Heat Shock Proteins. Stem Cell Reports.

[2] Andersen J, VanScoy S, Cheng TF, Gomez D, Reich NC (2008). IRF-3-dependent and augmented target genes during viral infection. Genes Immun.

[3] Andrade NS, Ramic M, Esanov R, Liu W, Rybin MJ, Gaidosh G, Abdallah A, Del'Olio S, Huff TC, Chee NT (2020). Dipeptide repeat proteins inhibit homology-directed DNA double strand break repair in C9ORF72 ALS/FTD. Mol Neurodegener.

[4] Arlotta P, Molyneaux BJ, Chen J, Inoue J, Kominami R, Macklis JD (2005). Neuronal subtype-specific genes that control corticospinal motor neuron development in vivo. Neuron.

[5] Bang J, Spina S, Miller BL (2015). Frontotemporal dementia. Lancet.

[6] Barber GN (2014). STING-dependent cytosolic DNA sensing pathways. Trends Immunol.

[7] Barmada SJ, Serio A, Arjun A, Bilican B, Daub A, Ando DM, Tsvetkov A, Pleiss M, Li X, Peisach D (2014). Autophagy induction enhances TDP43 turnover and survival in neuronal ALS models. Nat Chem Biol.

[8] Benussi A, Di Lorenzo F, Dell'Era V, Cosseddu M, Alberici A, Caratozzolo S, Cotelli MS, Micheli A, Rozzini L, Depari A (2017). Transcranial magnetic stimulation distinguishes Alzheimer disease from frontotemporal dementia. Neurology.

[9] Boulting GL, Kiskinis E, Croft GF, Amoroso MW, Oakley DH, Wainger BJ, Williams DJ, Kahler DJ, Yamaki M, Davidow L (2011). A functionally characterized test set of human induced pluripotent stem cells. Nat Biotechnol.

[10] Brown RH, Al-Chalabi A (2017). Amyotrophic Lateral Sclerosis. N Engl J Med.

[11] Chen Q, Sun L, Chen ZJ (2016). Regulation and function of the cGAS-STING pathway of cytosolic DNA sensing. Nat Immunol.

[12] Chen Y, Tristan CA, Chen L, Jovanovic VM, Malley C, Chu PH, Ryu S, Deng T, Ormanoglu P, Tao D (2021). A versatile polypharmacology platform promotes cytoprotection and viability of human pluripotent and differentiated cells. Nat Methods.

[13] Chew J, Cook C, Gendron TF, Jansen-West K, Del Rosso G, Daughrity LM, Castanedes-Casey M, Kurti A, Stankowski JN, Disney MD (2019). Aberrant deposition of stress granule-resident proteins linked to C9orf72-associated TDP-43 proteinopathy. Mol Neurodegener.

[14] Ciccia A, Elledge SJ (2010). The DNA damage response: making it safe to play with knives. Mol Cell.

[15] Collart MA, Baeuerle P, Vassalli P (1990). Regulation of tumor necrosis factor alpha transcription in macrophages: involvement of four kappa B-like motifs and of constitutive and inducible forms of NF-kappa B. Mol Cell Biol.

[16] Cook CN, Wu Y, Odeh HM, Gendron TF, Jansen-West K, Del Rosso G, Yue M, Jiang P, Gomes E, Tong J (2020). C9orf72 poly(GR) aggregation induces TDP-43 proteinopathy. Sci Transl Med.

[17] Decout A (2021). The cGAS–STING pathway as a therapeutic target in inflammatory diseases. Nat Rev Immunol.

[18] Dobbs N, Burnaevskiy N, Chen D, Gonugunta VK, Alto NM, Yan N (2015). STING Activation by Translocation from the ER Is Associated with Infection and Autoinflammatory Disease. Cell Host Microbe.

[19] Dobin A, Davis CA, Schlesinger F, Drenkow J, Zaleski C, Jha S, Batut P, Chaisson M, Gingeras TR (2013). STAR: ultrafast universal RNA-seq aligner. Bioinformatics.

[20] Dunphy G, Flannery SM, Almine JF, Connolly DJ, Paulus C, Jonsson KL, Jakobsen MR, Nevels MM, Bowie AG, Unterholzner L (2018). Non-canonical Activation of the DNA Sensing Adaptor STING by ATM and IFI16 Mediates NF-kappaB Signaling after Nuclear DNA Damage. Mol Cell.

[21] El-Khamisy SF (2011). To live or to die: a matter of processing damaged DNA termini in neurons. EMBO Mol Med.

[22] Farg MA, Konopka A, Soo KY, Ito D, Atkin JD (2017). The DNA damage response (DDR) is induced by the C9orf72 repeat expansion in amyotrophic lateral sclerosis. Hum Mol Genet.

[23] Fil D, DeLoach A, Yadav S, Alkam D, MacNicol M, Singh A, Compadre CM, Goellner JJ, O'Brien CA, Fahmi T (2017). Mutant Profilin1 transgenic mice recapitulate cardinal features of motor neuron disease. Hum Mol Genet.

[24] Gendron TF, Petrucelli L (2018). Disease Mechanisms of C9ORF72 Repeat Expansions. Cold Spring Harb Perspect Med.

[25] Giannini M, Bayona-Feliu A, Sproviero D, Barroso SI, Cereda C, Aguilera A (2020). TDP-43 mutations link Amyotrophic Lateral Sclerosis with R-loop homeostasis and R loop-mediated DNA damage. PLoS Genet.

[26] Gong T, Liu L, Jiang W, Zhou R (2020). DAMP-sensing receptors in sterile inflammation and inflammatory diseases. Nat Rev Immunol.

[27] Gonugunta VK, Sakai T, Pokatayev V, Yang K, Wu J, Dobbs N, Yan N (2017). Trafficking-Mediated STING Degradation Requires Sorting to Acidified Endolysosomes and Can Be Targeted to Enhance Anti-tumor Response. Cell Rep.

[28] Guerrero EN, Mitra J, Wang H, Rangaswamy S, Hegde PM, Basu P, Rao KS, Hegde ML (2019). Amyotrophic lateral sclerosis-associated TDP-43 mutation Q331K prevents nuclear translocation of XRCC4-DNA ligase 4 complex and is linked to genome damage-mediated neuronal apoptosis. Hum Mol Genet.

[29] Gui X, Yang H, Li T, Tan X, Shi P, Li M, Du F, Chen ZJ (2019). Autophagy induction via STING trafficking is a primordial function of the cGAS pathway. Nature.

[30] Gulen MF, Koch U, Haag SM, Schuler F, Apetoh L, Villunger A, Radtke F, Ablasser A (2017). Signalling strength determines proapoptotic functions of STING. Nat Commun.

[31] Haag SM, Gulen MF, Reymond L, Gibelin A, Abrami L, Decout A, Heymann M, van der Goot FG, Turcatti G, Behrendt R (2018). Targeting STING with covalent small-molecule inhibitors. Nature.

[32] Hammer Jr RP, Tomiyasu U, Scheibel AB (1979). Degeneration of the human Betz cell due to amyotrophic lateral sclerosis. Exp Neurol.

[33] Hartlova A, Erttmann SF, Raffi FA, Schmalz AM, Resch U, Anugula S, Lienenklaus S, Nilsson LM, Kroger A, Nilsson JA (2015). DNA damage primes the type I interferon system via the cytosolic DNA sensor STING to promote anti-microbial innate immunity. Immunity.

[34] Hawrot J, Imhof S, Wainger BJ (2020). Modeling cell-autonomous motor neuron phenotypes in ALS using iPSCs. Neurobiol Dis.

[35] Held A, Adler M, Marques C, Reyes CJ, Kavuturu AS, Quadros A, Ndayambaje IS, Lara E, Ward M, Lagier-Tourenne C (2023). iPSC motor neurons, but not other derived cell types, capture gene expression changes in postmortem sporadic ALS motor neurons. Cell Rep.

[36] Heneka MT, Kummer MP, Latz E (2014). Innate immune activation in neurodegenerative disease. Nat Rev Immunol.

[37] Hill SJ, Mordes DA, Cameron LA, Neuberg DS, Landini S, Eggan K, Livingston DM (2016). Two familial ALS proteins function in prevention/repair of transcription-associated DNA damage. Proc Natl Acad Sci U S A.

[38] Hiscott J, Marois J, Garoufalis J, D'Addario M, Roulston A, Kwan I, Pepin N, Lacoste J, Nguyen H, Bensi G (1993). Characterization of a functional NF-kappa B site in the human interleukin 1 beta promoter: evidence for a positive autoregulatory loop. Mol Cell Biol.

[39] Hopfner KP, Hornung V (2020). Molecular mechanisms and cellular functions of cGAS-STING signalling. Nat Rev Mol Cell Biol.

[40] Ishikawa H, Barber GN (2008). STING is an endoplasmic reticulum adaptor that facilitates innate immune signalling. Nature.

[41] Ishikawa H, Ma Z, Barber GN (2009). STING regulates intracellular DNA-mediated, type I interferon-dependent innate immunity. Nature.

[42] Kim BW, Jeong YE, Wong M, Martin LJ (2020). DNA damage accumulates and responses are engaged in human ALS brain and spinal motor neurons and DNA repair is activatable in iPSC-derived motor neurons with SOD1 mutations. Acta Neuropathol Commun.

[43] Kim SH, Engelhardt JI, Henkel JS, Siklos L, Soos J, Goodman C, Appel SH (2004). Widespread increased expression of the DNA repair enzyme PARP in brain in ALS. Neurology.

[44] Kisby GE, Milne J, Sweatt C (1997). Evidence of reduced DNA repair in amyotrophic lateral sclerosis brain tissue. NeuroReport.

[45] Kiskinis E, Sandoe J, Williams LA, Boulting GL, Moccia R, Wainger BJ, Han S, Peng T, Thams S, Mikkilineni S (2014). Pathways disrupted in human ALS motor neurons identified through genetic correction of mutant SOD1. Cell Stem Cell.

[46] Kok JR, Palminha NM, Dos Santos SC, El-Khamisy SF, Ferraiuolo L (2021). DNA damage as a mechanism of neurodegeneration in ALS and a contributor to astrocyte toxicity. Cell Mol Life Sci.

[47] Konopka A, Atkin JD (2018). The Emerging Role of DNA Damage in the Pathogenesis of the C9orf72 Repeat Expansion in Amyotrophic Lateral Sclerosis. Int J Mol Sci.

[48] Konopka A, Whelan DR, Jamali MS, Perri E, Shahheydari H, Toth RP, Parakh S, Robinson T, Cheong A, Mehta P (2020). Impaired NHEJ repair in amyotrophic lateral sclerosis is associated with TDP-43 mutations. Mol Neurodegener.

[49] Krach F, Batra R, Wheeler EC, Vu AQ, Wang R, Hutt K, Rabin SJ, Baughn MW, Libby RT, Diaz-Garcia S (2018). Transcriptome-pathology correlation identifies interplay between TDP-43 and the expression of its kinase CK1E in sporadic ALS. Acta Neuropathol.

[50] Krug L, Chatterjee N, Borges-Monroy R, Hearn S, Liao WW, Morrill K, Prazak L, Rozhkov N, Theodorou D, Hammell M (2017). Retrotransposon activation contributes to neurodegeneration in a Drosophila TDP-43 model of ALS. PLoS Genet.

[51] Kumar V (2019). Toll-like receptors in the pathogenesis of neuroinflammation. J Neuroimmunol.

[52] Kwon I, Xiang S, Kato M, Wu L, Theodoropoulos P, Wang T, Kim J, Yun J, Xie Y, McKnight SL (2014). Poly-dipeptides encoded by the C9orf72 repeats bind nucleoli, impede RNA biogenesis, and kill cells. Science.

[53] Lee H, Fenster RJ, Pineda SS, Gibbs WS, Mohammadi S, Davila-Velderrain J, Garcia FJ, Therrien M, Novis HS, Gao F (2020). Cell Type-Specific Transcriptomics Reveals that Mutant Huntingtin Leads to Mitochondrial RNA Release and Neuronal Innate Immune Activation. Neuron.

[54] Li F, Chen Y, Zhang Z, Ouyang J, Wang Y, Yan R, Huang S, Gao GF, Guo G, Chen JL (2015). Robust expression of vault RNAs induced by influenza A virus plays a critical role in suppression of PKR-mediated innate immunity. Nucleic Acids Res.

[55] Lin H-C, He Z, Ebert S, Schörnig M, Santel M, Nikolova MT (2021). Ngn2 induces diverse neuron types from human pluripotency. Stem Cell Reports.

[56] Lopez-Gonzalez R, Lu Y, Gendron TF, Karydas A, Tran H, Yang D, Petrucelli L, Miller BL, Almeida S, Gao FB (2016). Poly(GR) in C9ORF72-Related ALS/FTD Compromises Mitochondrial Function and Increases Oxidative Stress and DNA Damage in iPSC-Derived Motor Neurons. Neuron.

[57] Love MI, Huber W, Anders S (2014). Moderated estimation of fold change and dispersion for RNA-seq data with DESeq2. Genome Biol.

[58] Ma F, Li B, Yu Y, Iyer SS, Sun M, Cheng G (2015). Positive feedback regulation of type I interferon by the interferon-stimulated gene STING. EMBO Rep.

[59] Madabhushi R, Gao F, Pfenning AR, Pan L, Yamakawa S, Seo J, Rueda R, Phan TX, Yamakawa H, Pao PC (2015). Activity-Induced DNA Breaks Govern the Expression of Neuronal Early-Response Genes. Cell.

[60] Madabhushi R, Pan L, Tsai LH (2014). DNA damage and its links to neurodegeneration. Neuron.

[61] Marques C, Burg T, Scekic-Zahirovic J, Fischer M, Rouaux C (2021). Upper and Lower Motor Neuron Degenerations Are Somatotopically Related and Temporally Ordered in the Sod1 Mouse Model of Amyotrophic Lateral Sclerosis. Brain Sci.

[62] McCauley ME, O'Rourke JG, Yanez A, Markman JL, Ho R, Wang X, Chen S, Lall D, Jin M, Muhammad A (2020). C9orf72 in myeloid cells suppresses STING-induced inflammation. Nature.

[63] Menon P, Geevasinga N, Yiannikas C, Howells J, Kiernan MC, Vucic S (2015). Sensitivity and specificity of threshold tracking transcranial magnetic stimulation for diagnosis of amyotrophic lateral sclerosis: a prospective study. Lancet Neurol.

[64] Mitra J, Guerrero EN, Hegde PM, Liachko NF, Wang H, Vasquez V, Gao J, Pandey A, Taylor JP, Kraemer BC (2019). Motor neuron disease-associated loss of nuclear TDP-43 is linked to DNA double-strand break repair defects. Proc Natl Acad Sci U S A.

[65] Moakley D, Koh J, Pereira JD, DuBreuil DM, Devlin AC, Berezovski E, Zhu K, Wainger BJ (2019). Pharmacological Profiling of Purified Human Stem Cell-Derived and Primary Mouse Motor Neurons. Sci Rep.

[66] Molyneaux BJ, Arlotta P, Menezes JR, Macklis JD (2007). Neuronal subtype specification in the cerebral cortex. Nat Rev Neurosci.

[67] Mordes DA, Prudencio M, Goodman LD, Klim JR, Moccia R, Limone F, Pietilainen O, Chowdhary K, Dickson DW, Rademakers R (2018). Dipeptide repeat proteins activate a heat shock response found in C9ORF72-ALS/FTLD patients. Acta Neuropathol Commun.

[68] Motwani M, Pesiridis S, Fitzgerald KA (2019). DNA sensing by the cGAS-STING pathway in health and disease. Nat Rev Genet.

[69] Mullaart E, Boerrigter ME, Ravid R, Swaab DF, Vijg J (1990). Increased levels of DNA breaks in cerebral cortex of Alzheimer's disease patients. Neurobiol Aging.

[70] Nakahira K, Haspel JA, Rathinam VA, Lee SJ, Dolinay T, Lam HC, Englert JA, Rabinovitch M, Cernadas M, Kim HP (2011). Autophagy proteins regulate innate immune responses by inhibiting the release of mitochondrial DNA mediated by the NALP3 inflammasome. Nat Immunol.

[71] Naumann M, Pal A, Goswami A, Lojewski X, Japtok J, Vehlow A, Naujock M, Gunther R, Jin M, Stanslowsky N (2018). Impaired DNA damage response signaling by FUS-NLS mutations leads to neurodegeneration and FUS aggregate formation. Nat Commun.

[72] Nihei K, McKee AC, Kowall NW (1993). Patterns of neuronal degeneration in the motor cortex of amyotrophic lateral sclerosis patients. Acta Neuropathol.

[73] Nihei Y, Mori K, Werner G, Arzberger T, Zhou Q, Khosravi B, Japtok J, Hermann A, Sommacal A, Weber M (2020). Poly-glycine-alanine exacerbates C9orf72 repeat expansion-mediated DNA damage via sequestration of phosphorylated ATM and loss of nuclear hnRNPA3. Acta Neuropathol.

[74] Ou L, Zhang A, Cheng Y, Chen Y (2021). The cGAS-STING Pathway: A Promising Immunotherapy Target. Front Immunol.

[75] Pantazis CB, Yang A, Lara E, McDonough JA, Blauwendraat C, Peng L (2022). A reference human induced pluripotent stem cell line for large-scale collaborative studies. Cell Stem Cell.

[76] Paul BD, Snyder SH, Bohr VA (2021). Signaling by cGAS-STING in Neurodegeneration, Neuroinflammation, and Aging. Trends Neurosci.

[77] Pereira JD, DuBreuil DM, Devlin AC, Held A, Sapir Y, Berezovski E, Hawrot J, Dorfman K, Chander V, Wainger BJ (2021). Human sensorimotor organoids derived from healthy and amyotrophic lateral sclerosis stem cells form neuromuscular junctions. Nat Commun.

[78] Pessina F, Gioia U, Brandi O, Farina S, Ceccon M, Francia S, d'Adda di Fagagna F (2021). DNA Damage Triggers a New Phase in Neurodegeneration. Trends Genet.

[79] Philips TWR (2011). Neuroinflammation in amyotrophic lateral sclerosis: role of glial activation in motor neuron disease. Lancet Neurol.

[80] Qiu H, Lee S, Shang Y, Wang WY, Au KF, Kamiya S, Barmada SJ, Finkbeiner S, Lui H, Carlton CE (2014). ALS-associated mutation FUS-R521C causes DNA damage and RNA splicing defects. J Clin Invest.

[81] Robert T, Vanoli F, Chiolo I, Shubassi G, Bernstein KA, Rothstein R, Botrugno OA, Parazzoli D, Oldani A, Minucci S (2011). HDACs link the DNA damage response, processing of double-strand breaks and autophagy. Nature.

[82] Rodriguez S, Sahin A, Schrank BR, Al-Lawati H, Costantino I, Benz E, Fard D, Albers AD, Cao L, Gomez AC (2021). Genome-encoded cytoplasmic double-stranded RNAs, found in C9ORF72 ALS-FTD brain, propagate neuronal loss. Sci Transl Med.

[83] Rothstein JD (1990). Abnormal excitatory amino acid metabolism in amyotrophic lateral sclerosis. Ann of Neurol.

[84] Sasaki S, Iwata M (2000). Immunocytochemical and ultrastructural study of the motor cortex in patients with lower motor neuron disease. Neurosci Lett.

[85] Sauer JD, Sotelo-Troha K, von Moltke J, Monroe KM, Rae CS, Brubaker SW, Hyodo M, Hayakawa Y, Woodward JJ, Portnoy DA (2011). The N-ethyl-N-nitrosourea-induced Goldenticket mouse mutant reveals an essential function of Sting in the in vivo interferon response to Listeria monocytogenes and cyclic dinucleotides. Infect Immun.

[86] Schneider CA, Rasband WS, Eliceiri KW (2012). NIH Image to ImageJ: 25 years of image analysis. Nat Methods.

[87] Selvaraj BT, Livesey MR, Zhao C, Gregory JM, James OT, Cleary EM, Chouhan AK, Gane AB, Perkins EM, Dando O (2018). C9ORF72 repeat expansion causes vulnerability of motor neurons to Ca(2+)-permeable AMPA receptor-mediated excitotoxicity. Nat Commun.

[88] Shanbhag NM, Evans MD, Mao W, Nana AL, Seeley WW, Adame A, Rissman RA, Masliah E, Mucke L (2019). Early neuronal accumulation of DNA double strand breaks in Alzheimer's disease. Acta Neuropathol Commun.

[89] Sliter DA, Martinez J, Hao L, Chen X, Sun N, Fischer TD, Burman JL, Li Y, Zhang Z, Narendra DP (2018). Parkin and PINK1 mitigate STING-induced inflammation. Nature.

[90] Son YH, Jeong YT, Lee KA, Choi KH, Kim SM, Rhim BY, Kim K (2008). Roles of MAPK and NF-kappaB in interleukin-6 induction by lipopolysaccharide in vascular smooth muscle cells. J Cardiovasc Pharmacol.

[91] Suberbielle E, Sanchez PE, Kravitz AV, Wang X, Ho K, Eilertson K, Devidze N, Kreitzer AC, Mucke L (2013). Physiologic brain activity causes DNA double-strand breaks in neurons, with exacerbation by amyloid-beta. Nat Neurosci.

[92] Subramanian A, Tamayo P, Mootha VK, Mukherjee S, Ebert BL, Gillette MA, Paulovich A, Pomeroy SL, Golub TR, Lander ES (2005). Gene set enrichment analysis: a knowledge-based approach for interpreting genome-wide expression profiles. Proc Natl Acad Sci U S A.

[93] Takeda S, Wegmann S, Cho H, DeVos SL, Commins C, Roe AD, Nicholls SB, Carlson GA, Pitstick R, Nobuhara CK (2015). Neuronal uptake and propagation of a rare phosphorylated high-molecular-weight tau derived from Alzheimer's disease brain. Nat Commun.

[94] Tam OH, Rozhkov NV, Shaw R, Kim D, Hubbard I, Fennessey S, Propp N, Consortium NA, Fagegaltier D, Harris BT et al (2019) Postmortem Cortex Samples Identify Distinct Molecular Subtypes of ALS: Retrotransposon Activation, Oxidative Stress, and Activated Glia. Cell Rep 29: 1164–1177 10.1016/j.celrep.2019.09.066 PMC6866666 31665631

[95] Tigano M, Vargas DC, Tremblay-Belzile S, Fu Y, Sfeir A (2021). Nuclear sensing of breaks in mitochondrial DNA enhances immune surveillance. Nature.

[96] Vincent J, Adura C, Gao P, Luz A, Lama L, Asano Y, Okamoto R, Imaeda T, Aida J, Rothamel K (2017). Small molecule inhibition of cGAS reduces interferon expression in primary macrophages from autoimmune mice. Nat Commun.

[97] Wainger BJ, Macklin EA, Vucic S, McIlduff CE, Paganoni S, Maragakis NJ, Bedlack R, Goyal NA, Rutkove SB, Lange DJ (2021). Effect of Ezogabine on Cortical and Spinal Motor Neuron Excitability in Amyotrophic Lateral Sclerosis: A Randomized Clinical Trial. JAMA Neurol.

[98] Walker C, Herranz-Martin S, Karyka E, Liao C, Lewis K, Elsayed W, Lukashchuk V, Chiang SC, Ray S, Mulcahy PJ (2017). C9orf72 expansion disrupts ATM-mediated chromosomal break repair. Nat Neurosci.

[99] Wang WY, Pan L, Su SC, Quinn EJ, Sasaki M, Jimenez JC, Mackenzie IR, Huang EJ, Tsai LH (2013). Interaction of FUS and HDAC1 regulates DNA damage response and repair in neurons. Nat Neurosci.

[100] Wu CC, Jin LW, Wang IF, Wei WY, Ho PC, Liu YC, Tsai KJ (2020). HDAC1 dysregulation induces aberrant cell cycle and DNA damage in progress of TDP-43 proteinopathies. EMBO Mol Med.

[101] Wu CC, Li TK, Farh L, Lin LY, Lin TS, Yu YJ, Yen TJ, Chiang CW, Chan NL (2011). Structural basis of type II topoisomerase inhibition by the anticancer drug etoposide. Science.

[102] Yu CH, Davidson S, Harapas CR, Hilton JB, Mlodzianoski MJ, Laohamonthonkul P, Louis C, Low RRJ, Moecking J, De Nardo D (2020). TDP-43 Triggers Mitochondrial DNA Release via mPTP to Activate cGAS/STING in ALS. Cell.

[103] Yusa K, Zhou L, Li MA, Bradley A, Craig NL (2011). A hyperactive piggyBac transposase for mammalian applications. Proc Natl Acad Sci U S A.

[104] Zhang Y, Pak C, Han Y, Ahlenius H, Zhang Z, Chanda S, Marro S, Patzke C, Acuna C, Covy J (2013). Rapid single-step induction of functional neurons from human pluripotent stem cells. Neuron.

[105] Zhang YJ, Guo L, Gonzales PK, Gendron TF, Wu Y, Jansen-West K, O'Raw AD, Pickles SR, Prudencio M, Carlomagno Y (2019). Heterochromatin anomalies and double-stranded RNA accumulation underlie C9orf72 poly(PR) toxicity. Science.

[106] Ziff OJ, Neeves J, Mitchell J, Tyzack G, Martinez-Ruiz C, Luisier R, Chakrabarti AM, McGranahan N, Litchfield K, Boulton SJ (2023). Integrated transcriptome landscape of ALS identifies genome instability linked to TDP-43 pathology. Nat Commun.

