# Supplementary material for: Neuronal STING activation in amyotrophic lateral sclerosis and frontotemporal dementia
Source: Acta Neuropathol. 2024 Mar 13;147(1):56. doi: 10.1007/s00401-024-02688-z (PMC10937762; doi:10.1007/s00401-024-02688-z)
Supplement: Supplementary file 1 — Supplementary file1 (PDF 10990 KB) [file 401_2024_2688_MOESM1_ESM.pdf]

Neuronal STING activation in amyotrophic lateral sclerosis and frontotemporal dementia

Christine Marques[1,2], Aaron Held[1,2], Katherine Dorfman[1], Joon Sung[1], Catherine Song[1], Amey S. Kavuturu[1], Corey Aguilar[1], Tommaso Russo[1], Derek H. Oakley[2,3], Mark W. Albers[1,2,4], Bradley T. Hyman[1,2,4], Leonard Petrucelli[5], Clotilde Lagier-Tourenne[1,2,6], *Brian J. Wainger[1,2,6,7,8]

[1]Department of Neurology, Sean M. Healey & AMG Center for ALS, Massachusetts General Hospital, Boston, MA, USA
[2]Harvard Medical School, Boston, MA, USA
[3]Department of Pathology, Massachusetts General Hospital, Harvard Medical School, Boston, MA, USA
[4]Alzheimer Disease Research Unit, Department of Neurology, Massachusetts General Hospital, Charlestown, MA, USA, Harvard Medical School, Boston, MA, USA.
[5]Department of Neuroscience, Mayo Clinic, Jacksonville, FL, USA
[6]Broad Institute of Harvard University and MIT, Cambridge, MA, USA
[7]Department of Anesthesiology, Critical Care and Pain Medicine, Massachusetts General Hospital, Boston, MA, USA
[8]Harvard Stem Cell Institute, Cambridge, MA, USA

***Corresponding author:** Brian J. Wainger, brian.wainger@mgh.harvard.edu.

Supplementary Figures and legends

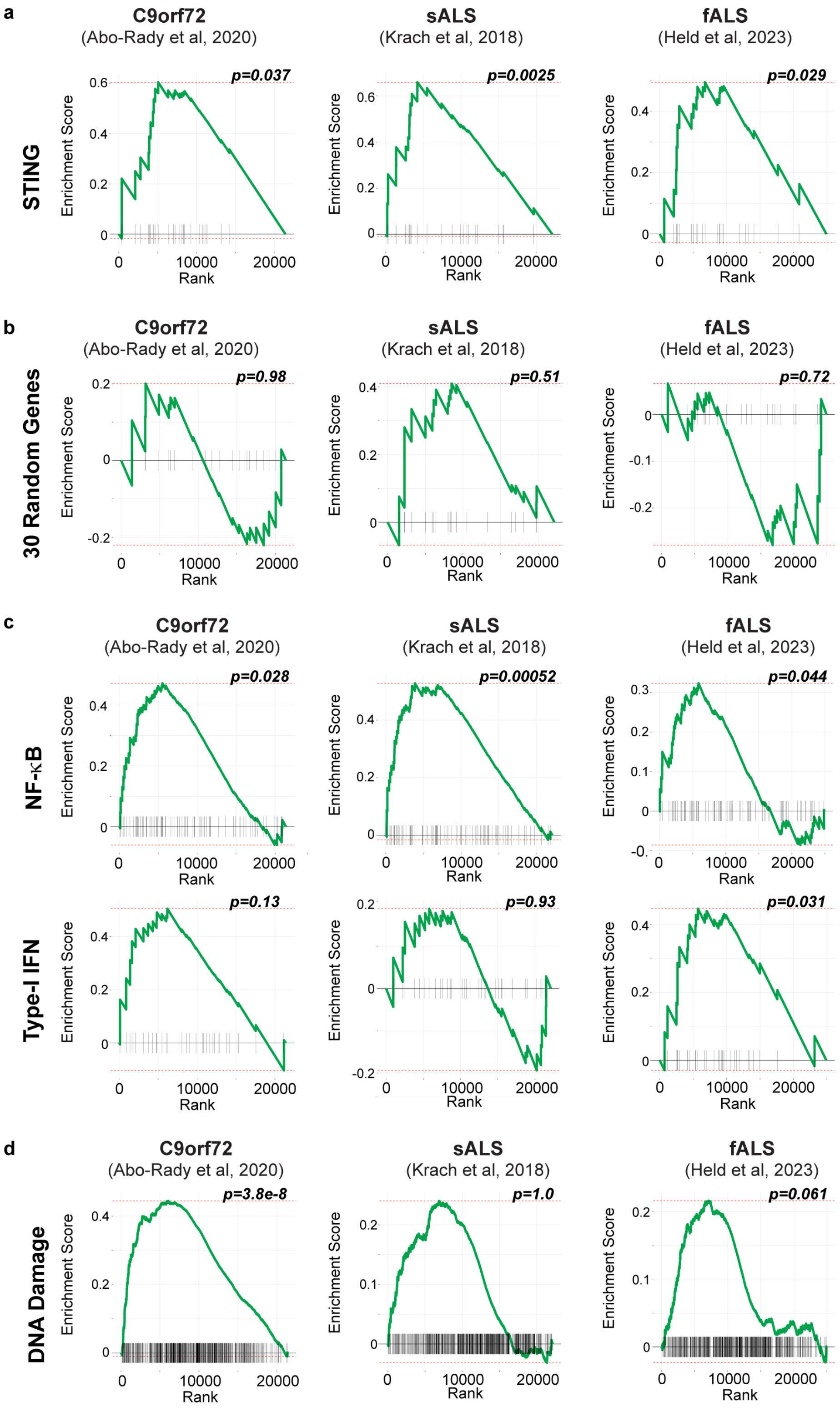

Fig. S1. Legend in the next page.

Fig. S1. Unbiased analysis of publicly available human SMN RNA-seq datasets suggests neuronal activation of the STING pathway and related upstream and downstream pathways in ALS.

Gene Set Enrichment Analysis (GSEA) score curves for pathways in three previously published ALS SMN RNAseq datasets: Abo-Rady et al., 2020 (C9orf72 vs isogenic control, left column), Krach et al., 2018 (laser captured SMNs from postmortem sALS vs matched control spinal cords, middle column), and Held et al., 2023 (SMNs generated from fALS iPSCs vs isogenic controls, right column) for 25 genes in the STING pathway **(a),** 30 random genes (negative control) **(b),** downstream pathways NF-κB-response genes (GO:0007249) (**c,** above) and type-I interferon response genes (GO:0032606) **(c,** below), and upstream DNA damage pathway (GO:0140612) **(d)**.

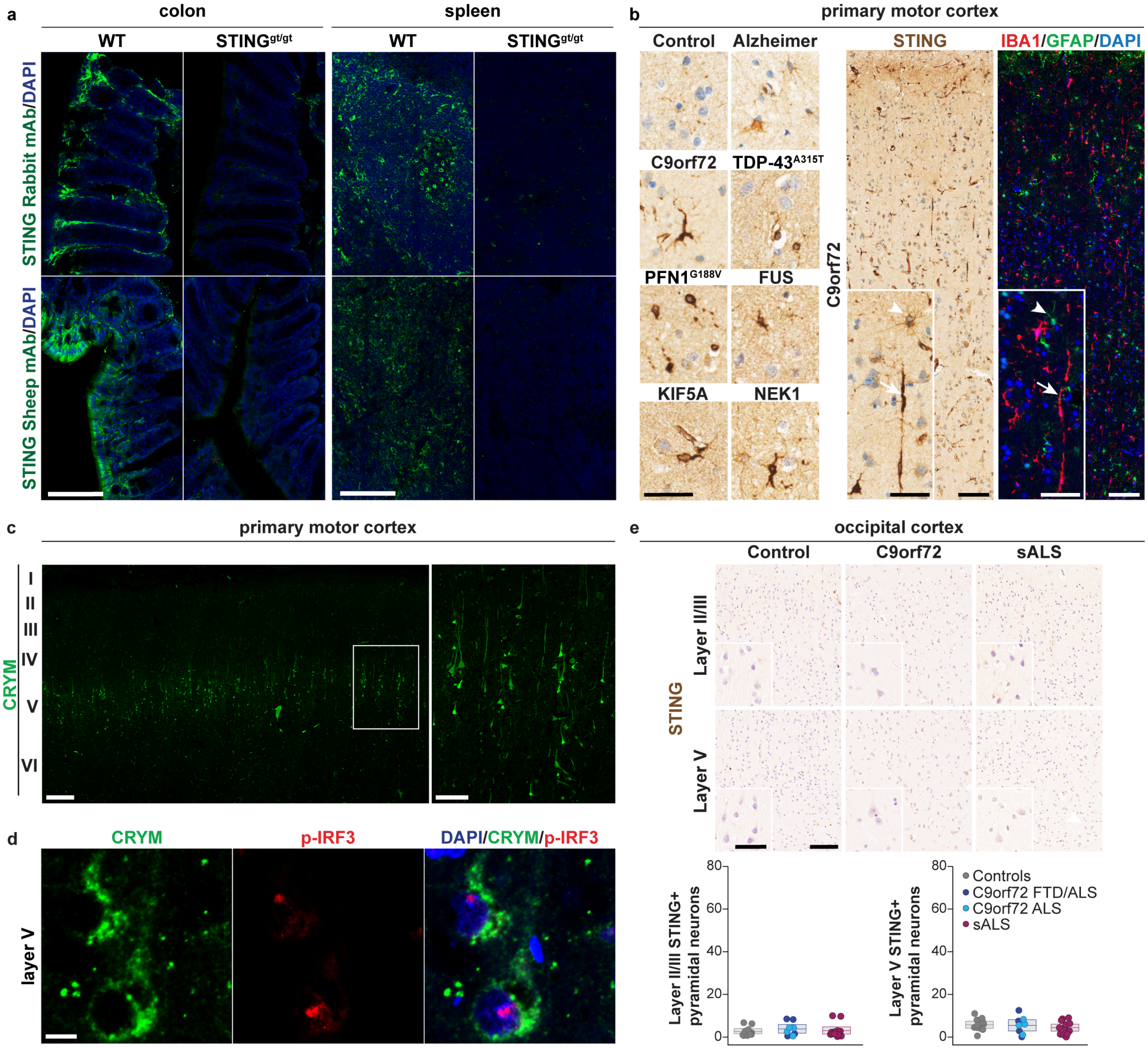

Fig. S2. Legend in the next page.

Fig. S2. STING is activated in glia as well as neurons of human ALS motor cortex but not in neurons from ALS occipital cortex. **a** Validation of the specificity of the two antibodies (STING sheep mAb, AF6516; STING Rabbit mAb, D2P2F) used in the study in colon and spleen sections (expressing high level of STING) of wild-type (WT) and goldenticket (gt) $STING^{gt/gt}$, which do not produce the protein (negative control). Scale bar = 100 µm. **b** Left, immunoperoxidase staining showing STING protein in glial cells from ALS, AD and matched non-neurological primary motor cortex. Right, immunofluorescence in C9orf72 human postmortem motor cortex with the microglial marker IBA1 (red), and astrocytic marker GFAP (green). Scale bars = 100 µm and 40 µm for inserts. **c** Immunofluorescence in primary motor cortex of non-neurological control brains with Mu-crystallin (CRYM) (green). Scale bars = 100 µm and 40 µm for inserts. **d** Co-immunofluorescence in primary motor cortex of a C9orf72 brain showing CRYM (green), p-IRF3 (red) and DAPI (blue) in layer V pyramidal neurons. Scale bar =10 µm. **e** Above, immunoperoxidase staining showing absence of STING protein in both layer II/III and layer V pyramidal neurons from the occipital cortex of C9orf72, sALS and matched non-neurological control brains. Below, boxplots depicting the number of layer II/III (left) and layer V (right) STING-positive pyramidal neurons in the occipital cortex of C9orf72 (3 FTD/ALS dark blue dots and 3 ALS light blue dots), sALS (n=12, purple), compared to non-neurological control (n=11, gray) brains. Scale bars = 100 µm and 40 µm for inserts. All data are shown as mean ± s.e.m, unpaired two-tailed Student's t-test: $p<0.05$*; $p<0.01$ **; $p<0.001$ ***.

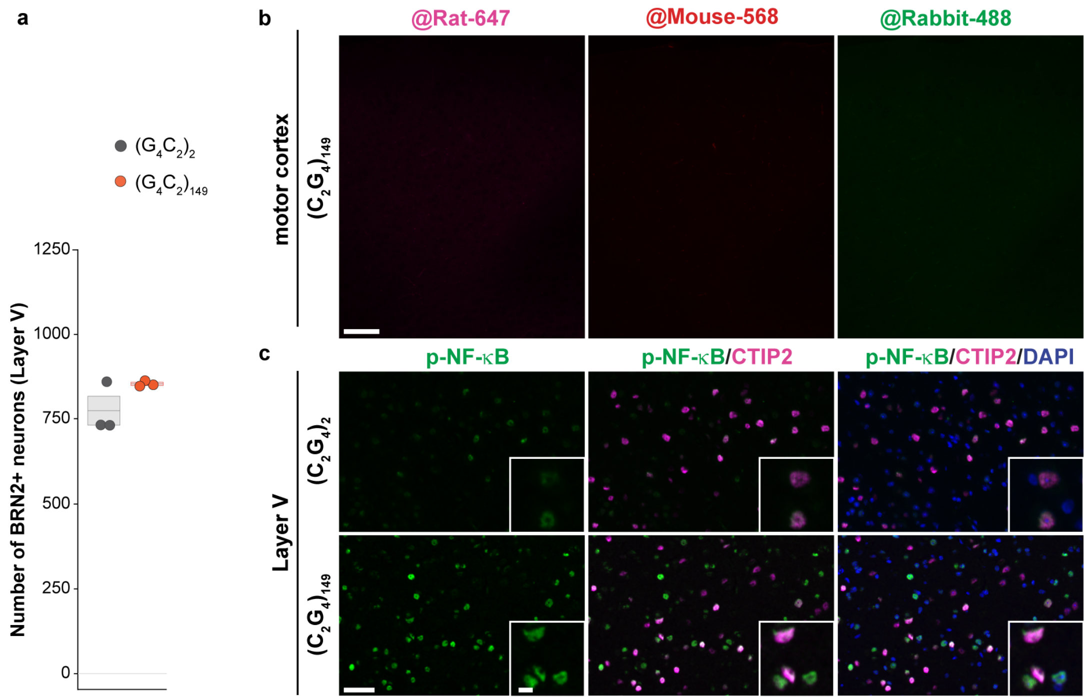

Fig. S3. STING downstream pathway activation, including p-NF-κB, is greater in CTIP2-positive layer V neurons from $(G_4C_2)_{149}$ compared to $(G_4C_2)_2$ mouse primary motor cortex.

a Boxplots depicting the number of layer II/III BRN2-positive neurons in the motor cortex of $(G4C2)_2$ (n=3, gray) and $(G4C2)_{149}$ (n=3, orange) mice. **b** Representative images of negative control immunostaining, using secondary antibodies only, on the motor cortex of $(G_4C_2)_{149}$ mice demonstrate absence of non-specific binding due to dipeptide repeats aggregates. **c** Representative immunofluorescence images of p-NF-κB (green), CTIP2 (magenta), and DAPI (blue) in layer V of the motor cortex of $(G_4C_2)_{149}$ compared to $(G_4C_2)_2$ control mice. Bottom right insert in each picture shows high magnification view. Scale bars = 50 µm and 10 µm for inserts.

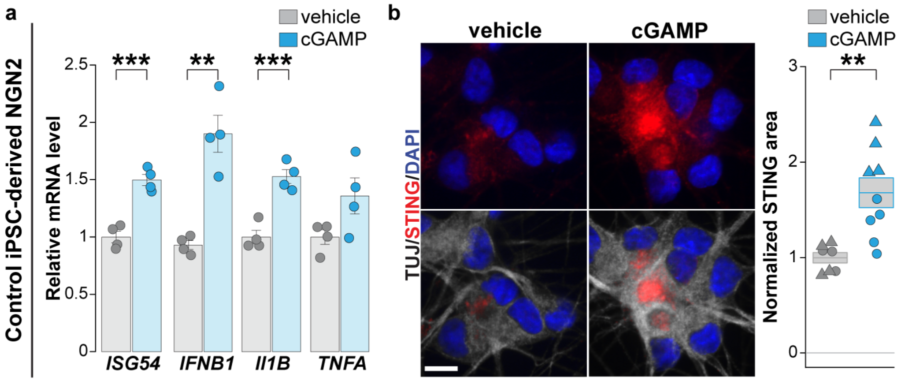

Fig. S4. STING pathway is present and functional within control human iPSC-derived NGN2 neurons.

a RT-qPCR analysis of the downstream canonical IRF3 (*ISG54*, *IFNB1*) and non-canonical NF-κB (*IL1B*, *TNFA*) response gene expression in control iPSC-derived NGN2 neurons after vehicle (gray) or the STING agonist cGAMP treatment (blue, 20 µM, 4h). Each dot represents an independent differentiation (n=4). **b** Left, representative immunofluorescence staining of STING (red) in control iPSC-derived NGN2 neurons treated with vehicle control or cGAMP (20 µM, 4h). Right, quantification of cytoplasmic STING area (per well/per cell) in wells treated with vehicle (gray) or cGAMP (blue). Each object represents a well, and each symbol represents an independent experiment (triangles, circles). Scale bar = 10 µm. Data are shown as mean ± s.e.m, unpaired two-tailed Student's t-test: $p<0.05$*; $p<0.01$ **; $p<0.001$ ***.

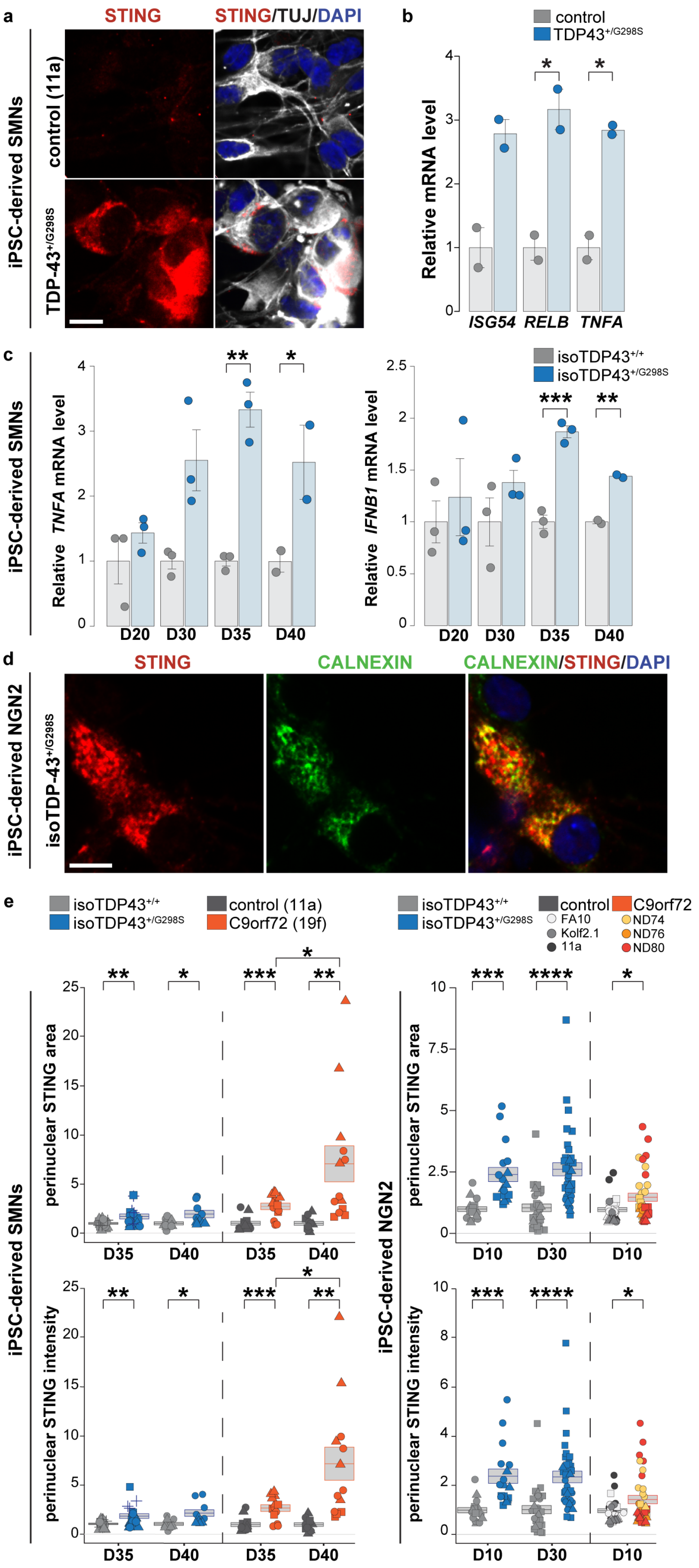

Fig. S5. legend next page.

Fig. S5 Activated neuronal STING RNA profiles and perinuclear accumulation in human ALS iPSC-derived neurons.

a Immunofluorescence staining for STING (red), TUJ1 (white), DAPI (blue) in iPSC-derived SMNs at day 35 of differentiation from a distinct TDP-43$^{+/G298S}$ ALS subject compared to healthy control (11a). **b** RT-qPCR analysis of downstream innate immune response genes in control 11a (gray) and TDP-43$^{+/G298S}$ ALS subject (blue) iPSC-derived SMNs at day 35. **c** RT-qPCR analysis of downstream innate immune response genes (*TNFA*, left; *IFNB1*, right) in control isoTDP-43$^{+/+}$ (gray) and isoTDP-43$^{+/G298S}$ (blue) over time (from D20 to D40). Each dot represents an independent differentiation (n=3; **b,c**). **d** Representative immunofluorescence images of STING protein (red) and calnexin (ER marker, green) in isoTDP-43$^{+/G298S}$ NGN2 neurons at day 10 of differentiation. **e** Quantification of perinuclear STING area (above) and perinuclear STING intensity (below) over time in isoTDP-43$^{+/+}$ control (gray) compared to isoTDP-43$^{+/G298S}$ (blue) as well as control (11a, gray) compared to C9orf72 (19f, orange dots) iPSC derived-SMNs (left) and iPSC-derived NGN2 neurons (right). NGN2 control versus C9orf72 comparison included NGN2 neurons from three independent control lines (gradient of gray: light, FA10; medium, Kolf2.1; dark, 11a) and three different iPSC-derived C9orf72 lines (gradient color: yellow, ND74; orange, ND76; red, ND80). Each object represents a well, and each symbol represents an independent differentiation (triangle, circle, crosses, squares). Scale bar = 10 μm. All data are shown as mean ± s.e.m, unpaired two-tailed Student's t-test: $p<0.05$*; $p<0.01$ **; $p<0.001$ ***.

Supplementary Tables

Supplementary Table 1. Human postmortem cases were used in this study.

Groups	Brain Bank	Case ID	Age at Death (years)	Sex	Post-mortem delay (hours)	Braak Stage	Disease diagnosis and genetic status	Tissue(s) analyzed
controls	**MADRC**	1721	87	Male	24	I	Control	Motor cortex/ occipital cortex/ Spinal cord
	**MADRC**	2339	90	Male	86	I	Control	Motor cortex/ occipital cortex
	**MADRC**	2327	91	Male	36	II	Control	Motor cortex/ occipital cortex
	**MADRC**	2392	56	Female	N/A	0	Control	Motor cortex/ occipital cortex
	**MADRC**	1346	57	Female	N/A	N/A	Control	Motor cortex/ occipital cortex
	**MADRC**	690	82	Female	N/A	N/A	Control	Motor cortex/ occipital cortex
	**MADRC**	1314	56	Male	N/A	Not Done	Control	Motor cortex/ occipital cortex
	**MADRC**	803	51	Male	N/A	N/A	Control	Motor cortex/ occipital cortex
	**MADRC**	692	56	Male	N/A	N/A	Control	Motor cortex/ occipital cortex/ Spinal cord
	**MADRC**	569	55	Male	N/A	N/A	Control	occipital cortex/ Spinal cord
	**MADRC**	1982	77	Female	72	I	Control	Motor cortex/ occipital cortex
	**MADRC**	1837	68	Male	27	I	Control	Motor cortex/ Spinal cord
	**MADRC**	2405	76	Female	17	III	Control	Motor cortex
	**MADRC**	1965	76	Female	39	I	Control	Motor cortex
	**MADRC**	2018	90	Female	24	0	Control	Spinal cord
	**MADRC**	1886	58	Female	18	0	Control	Spinal cord
Alzheimer	**MADRC**	2428	77	Female	28	VI	Alzheimer's Disease Neuropathological Changes	Motor cortex
	**MADRC**	2389	77	Male	24	VI	Alzheimer's Disease Neuropathological Changes	Motor cortex

	**MADRC**	2367	77	Female	32	IV	Alzheimer's Disease Neuropathological Changes	Motor cortex
	**MADRC**	2308	81	Male	28	VI	Alzheimer's Disease Neuropathological Changes	Motor cortex
	**MADRC**	2267	70	Female	24	VI	Alzheimer's Disease Neuropathological Changes	Motor cortex
	**MADRC**	2247	78	Female	28	VI	Alzheimer's Disease Neuropathological Changes	Motor cortex
	**MADRC**	2399	74	Male	10	VI	Alzheimer's Disease Neuropathological Changes	Motor cortex
	**MADRC**	2364	75	Male	4	VI	Alzheimer's Disease Neuropathological Changes	Motor cortex
	**MADRC**	1955	69	Female	4	VI	Alzheimer's Disease Neuropathological Changes	Motor cortex
**C9orf72**	**MADRC**	2425	62	Female	17	0	ALS - FTLD - C9RF72	Motor cortex/ occipital cortex/ Spinal cord
	**MADRC**	2368	55	Female	72	0	ALS - C9orf72	Motor cortex/ occipital cortex/ Spinal cord
	**MADRC**	1844	51	Female	48	none	ALS - C9orf72	Motor cortex/ occipital cortex/ Spinal cord
	**MADRC**	1700	51	Female	15	none	ALS - FTLD - C9orf72	Motor cortex/ occipital cortex/ Spinal cord
	**MADRC**	1699	67	Female	19	0	ALS - C9orf72	Motor cortex/ occipital cortex/ Spinal cord

	**MADRC**	2269	68	Male	24	0	ALS - FTLD - C9orf72	Motor cortex/ occipital cortex/ Spinal cord
	**MADRC**	2036	61	Male	22	none	ALS - C9orf72	Motor cortex/ occipital cortex/ Spinal cord
	**MADRC**	2022	56	Male	20	N/A	ALS - C9orf72	Spinal cord
**sALS**	**MADRC**	2228	63	Male	N/A	0	ALS - Sporadic	Motor cortex/ occipital cortex/ Spinal cord
	**MADRC**	2093	57	Female	N/A	0	ALS - Sporadic	Motor cortex/ occipital cortex/ Spinal cord
	**MADRC**	2087	57	Male	N/A	0	ALS - Sporadic	Motor cortex/ occipital cortex/ Spinal cord
	**MADRC**	2007	87	Female	N/A	II	ALS - Sporadic	Motor cortex/ occipital cortex/ Spinal cord
	**MADRC**	1914	58	Male	N/A	0	ALS - Sporadic	Motor cortex/ occipital cortex/ Spinal cord
	**MADRC**	1804	64	Female	N/A	I	ALS - Sporadic	Motor cortex/ occipital cortex/ Spinal cord
	**MADRC**	1708	57	Male	N/A	II	ALS - Sporadic	Motor cortex/ occipital cortex/ Spinal cord
	**MADRC**	1441	73	Female	N/A	I	ALS - Sporadic	Motor cortex/ occipital cortex/ Spinal cord
	**MADRC**	1597	51	Male	N/A	N/A	ALS - Sporadic	Motor cortex/ occipital cortex/ Spinal cord
	**MADRC**	2008	70	Female	N/A	0	ALS - Sporadic	Motor cortex/ occipital cortex/ Spinal cord
	**MADRC**	1370	70	Female	N/A	I	ALS - Sporadic	Motor cortex/ occipital cortex
	**MADRC**	1823	81	Male	N/A	0	ALS - Sporadic	Motor cortex
	**MADRC**	2246	57	Female	N/A	0	ALS - Sporadic	Spinal cord
	**MADRC**	2469	72	Male	82	N/A	ALS - Sporadic	Spinal cord
	**MADRC**	2486	67	Male	31	II	ALS - Sporadic	Spinal cord

	**MADRC**	2418	62	Female	15	0	ALS - Sporadic	Spinal cord
**controls**	**VABBB**	VAB T100 017	70	Male	56	none	Control	Motor cortex/ Spinal cord
	**VABBB**	VAB T100 021	78	Male	27	none	Control	Motor cortex/ Spinal cord
	**VABBB**	VAB T090 015	66	Male	84	none	Control	Motor cortex/ Spinal cord
	**VABBB**	VAB T090 018	82	Male	50	none	Control	Motor cortex
**fALS**	**VABBB**	VAB T080 029	71	Male	65	none	ALS - NEK1:NM_001199400:exon11:c.C959A:p.A320E; NEK1:NM_012224:exon11:c.C959A:p.A320E;NEK1:NM_001199397:exon12:c.C959A:p.A320E;NEK1:NM_001199398:exon12:c.C959A:p.A320E;NEK1:NM_001199399:exon12:c.C959A:p.A320E	Motor cortex/ Spinal cord
	**VABBB**	VAB T100 024	63	Male	51	none	ALS - TARDBP:NM_007375:exon6:c.G943A:p.A315T	Motor cortex/ Spinal cord
	**VABBB**	VAB T110 011	83	Male	40	none	ALS - TARDBP	Motor cortex/ Spinal cord

	**VABBB**	VABT AZ14002 1	63	Male	41	none	ALS - FUS:NM_001170634:exon15:c.G1559A:p.R520H,FUS:NM_001170937:exon15:c.G1550A:p.R517H,FUS:NM_004960:exon15:c.G1562A:p.R521H	Motor cortex/ Spinal cord
	**VABBB**	VABT110 010	55	Male	39	none	ALS - PFN1:NM_005022:exon3:c.G353T:p.G118V	Motor cortex
	**VABBB**	VABT100 008	64	Male	46	none	ALS - KIF5	Motor cortex

MADRC: Massachusetts Alzheimer's disease research center
VABBB: Veterans Affairs Biorepository Brain Bank

Supplementary Table 2. Antibodies.

Antibodies	Manufacturer	Catalog number	Dilution
Rabbit TMEM173/STING antibody	proteintech	19851-1-AP	1/100
Sheep STING antibody	R&D Systems	AF6516	1/50
Rabbit p-IRF-3 (Ser396) (D6O1M) antibody	cell signaling Technology	29047S	1/50
Mouse p-NF-κB antibody	santa cruz biotechnology	sc-136548	1/50
Mouse Calnexin antibody (AF18)	Novus Biologicals	NB300-518	1/50
Rabbit Mu-crystallin antibody	abcam	ab220085	1/250
Mouse HuC/HuD antibody	sigma-aldrich	MABN153	1/2000
Chicken TUJ1 antibody	Aves Lab	TUJ	1/250
Rat CTIP2 antibody	abcam	ab18465	1/100
Rabbit p-Histone H2A.X (Ser139) (20E3) antibody	Cell Signaling Technology	9718T	1/50
Rabbit Brn2/POU3F2 (D2C1L) antibody	Cell Signaling Technology	12137	1/200
Goat Iba1 antibody	abcam	ab5076	1/1000
Rabbit Glial Fibrillary Acidic Protein antibody	Sigma-Aldrich	G9269	1/1000
Donkey anti-mouse 568	Thermofisher Scientific	A10037	1/500
Donkey anti-rabbit 488	Thermofisher Scientific	A21206	1/500
Donkey anti-rabbit 568	Thermofisher Scientific	A10042	1/500
Donkey anti-rabbit 647	Thermofisher Scientific	A31573	1/500
Donkey anti-sheep 647	Invitrogen	A21448	1/500
Donkey anti-Chicken 488	Jackson Immunoresearch	703-545-155	1/500
Goat anti-rat Cy3	abcam	ab98416	1/500
Donkey anti-rat 647	Thermofisher Scientific	A78947	1/500
Peroxidase AffiniPure Donkey anti-sheep IgG (H+L)	Jackson Immunoresearch	713-035-003	1/500

Supplementary Table 3. RT-qPCR Primer List.
Primers are divided in 2 groups: mouse and human.

Species	Primer name	Sequence (5'-3')
mouse	m-Hprt_F	TGAAGTACTCATTATAGTCAAGGGCA
	m-Hprt_R	CTGGTGAAAAGGACCTCTCG
	m-Tbp_F	CCAATGACTCCTATGACCCCTA
	m-Tbp_R	CAGCCAAGATTCACGGTAGAT
	m-Sting_F	GGTCACCGCTCCAAATATGTAG
	m-Sting_R	CAGTAGTCCAAGTTCGTGCGA
	m-Tnfa_F	CCAAATGGCCTCCCTCTCAT
	m-Tnfa_R	TGGTGGTTTGCTACGACGTG
	m-Il1b_F	TTCAGGCAGGCAGTATCACTC
	m-Il1b_R	GAAGGTCCACGGGAAAGACAC
	m-Il6_F	TAGTCCTTCCTACCCCAATTTCC
	m-Il6_R	TTGGTCCTTAGCCACTCCTTC
	m-Ifnb1_F	AGCTCCAAGAAAGGACGAACAT
	m-Ifnb1_R	GCCCTGTAGGTGAGGTTGATCT
	m-Ifna_F	GCAATCCTCCTAGACTCACTTCTGCA
	m-Ifna_R	TATAGTTCCTCACAGCCAGCAG
	m-Cxcl10_F	CGATGACGGGCCAGTGAGAATG
	m-Cxcl10_R	TCAACACGTGGGCAGGATAGGCT
human	h-HPRT_F	TCAGGCAGTATATCCAAAGATGGT
	h-HPRT_R	AGTCTGGCTTATATCCAACACTTCG
	h-GAPDH_R	GGGGTCATTGATGGCAACAATA
	h-GAPDH_F	ATGGGGAAGGTGAAGGTCG
	h-TNFA_F	TCTCTCAGCTCCACGCCATT
	h-TNFA_R	CCCAGGCAGTCAGATCATCTTC
	h-IFNB1_F	TGTCGCCTACTACCTGTTGTGC
	h-IFNB1_R	AACTGCAACCTTTCGAAGCC
	h-IFNA_F	AATGACAGAATTCATGAAAGCGT
	h-IFNA_R	GGAGGTTGTCAGAGCAGA
	h-ISG54_F	ACGGTATGCTTGGAACGATTG
	h-ISG54_R	AACCCAGAGTGTGGCTGATG
	h-CXCL10_F	GTGGCATTCAAGGAGTACCTC
	h-CXCL10_R	TGATGGCCTTCGATTCTGGATT
	h-IL1B_F	CCAGGGACAGGATATGGAGCA
	h-IL1B_R	TTCAACACGCAGGACAGGTACAG
	h-RELB_F	CATTGAGCGGAAGATTCAAC
	h-RELB_R	GCAGCTCTGATGTGTTTGTG
